# Characterization of Metabolites and Transcripts Involved in Flower Pigmentation in *Primula vulgaris*

**DOI:** 10.3389/fpls.2020.572517

**Published:** 2020-11-20

**Authors:** Long Li, Jing Ye, Houhua Li, Qianqian Shi

**Affiliations:** ^1^College of Forestry, Northwest A&F University, Yangling, China; ^2^College of Landscape Architecture and Art, Northwest A&F University, Yangling, China

**Keywords:** flavonoid biosynthetic pathway, carotenoids biosynthetic pathway, various colors, *Primula vulgaris*, MYB

## Abstract

*Primula vulgaris* exhibits a wide range of flower colors and is a valuable ornamental plant. The combination of flavonols/anthocyanins and carotenoids provides various colorations ranging from yellow to violet-blue. However, the complex metabolic networks and molecular mechanisms underlying the different flower colors of *P. vulgaris* remain unclear. Based on comprehensive analysis of morphological anatomy, metabolites, and gene expression in different-colored flowers of *P. vulgaris*, the mechanisms relating color-determining compounds to gene expression profiles were revealed. In the case of *P. vulgaris* flower color, hirsutin, rosinin, petunidin-, and cyanidin-type anthocyanins and the copigment herbacetin contributed to the blue coloration, whereas peonidin-, cyandin-, and delphinidin-type anthocyanins showed high accumulation levels in pink flowers. The color formation of blue and pink were mainly via the regulation of *F3′5′H* (*c53168*), *AOMT* (c47583, *c44905*), and *3GT* (*c50034*). Yellow coloration was mainly due to gossypetin and carotenoid, which were regulated by *F3H* (*c43100*), *F3 1* (*c53714*), *3GT* (*c53907*) as well as many carotenoid biosynthetic pathway-related genes. Co-expression network and transient expression analysis suggested a potential direct link between flavonoid and carotenoid biosynthetic pathways through MYB transcription factor regulation. This work reveals that transcription changes influence physiological characteristics, and biochemistry characteristics, and subsequently results in flower coloration in *P. vulgaris.*

## Introduction

*Primula*, a genus of ornamental perennial herbs, is one of the three most important garden plants because of the large number of varieties grown and the high income generated by the horticultural industry worldwide, and only *Rhododendron* and *Rosa* can compare with it (Richards, 1993). Moreover, *Primula* is well known as a flowering potted plant or bedding plant during winter and early spring due to its long blooming period, profuse flowering, high adaptability, and bright flower colors, especially their distinctive yellow and blue colors. *Primula vulgaris*, a common European primrose, has a broad range of flower colors including yellow, white, pink, blue, red, and purple. To date, cultivars of different colors have been produced by conventional breeding, including dark red, bright red, dark pink, deep yellow, blue, deep violet, light red, yellowish pink, red-rimmed white, light pink, and black flowers, and they are currently available on the market (Karlsson, 2002). These flower colors are largely determined by the spatially and temporally restricted deposition of varieties of anthocyanins, flavonols, and carotenoids (Harborne, 1968; Freyre and Griesbach, 2004; Quintana et al., 2007; Yamamizo et al., 2011). Malvidin-type anthocyanin contributes to blue flowers, delphinidin-type anthocyanin to red flowers, malvidin- and delphinidin-type anthocyanins to lilac and violet flowers, and pelargonidin-type anthocyanin to orange flowers in *Anagallis monelli* (Freyre and Griesbach, 2004; Yamamizo et al., 2011), while the carotenoid content determines the brightness of the yellow color in *P. vulgaris* (Cao and Liang, 2008).

The flavonoid biosynthetic pathway is well understood (Grotewold, 2006; Xu et al., 2015; Zhao and Tao, 2015) and can be divided into three stages: the first stage is the conversion of phenylalanine to coumaroyl-CoA by phenylalanine ammonia lyase (PAL), cinnamate-4-hydroxylase (C4H), and 4-coumarate CoA ligase (4CL), which is consistent with many secondary metabolism pathways; the second stage is the synthesis of dihydroflavonol from one molecule of coumaroyl-CoA and three molecules of malonyl-CoA catalyzed by many enzymes, such as chalcone synthase (CHS), chalcone isomerase (CHI), flavanone 3-hydroxylase (F3H), flavonoid 3*′*-hydroxylase (F3*′*H), flavonoid 3*′*5*′*-hydroxylase (F3*′*5*′*H), flavonol synthase (FLS), and flavone synthase (FNS); and the third stage is the synthesis of leucoanthocyanidins from dihydroflavonol by the action of dihydroflavonol 4-reductase (DFR) and then the synthesis of the corresponding colored anthocyanidins by anthocyanidin synthase (ANS) (Grotewold, 2006; Xu et al., 2015; Zhao and Tao, 2015). Subsequently, a series of modifications of anthocynidins are catalyzed by flavonoid glucosyltransferase (UFGT) and anthocyanin *O*-methyltransferase (AOMT) to form stable anthocynins (Yoshikazu et al., 2008; Lou et al., 2014). In this biosynthetic process, hirsutin, malvidin, and petunidin are formed from delphinidin by a series of methylation and glycosylation reactions to produce blue colors, while peonidin- and rosinin- based anthocyanins are converted from cyanidin through a series of glycosylation and methylation reactions to produce pink colors (Yoshikazu et al., 2008; Lou et al., 2014). Among these, rosinin is a lesser-known anthocyanidin methylated at the 7 position and glycosylated at the 3 and 5 positions of peonidin, and hirstidin is also a rare anthocyanidin, which is formed from delphinidin by methylating at the 3′, 5′, and 7 positions and glycosylating at the 3 and 5 positions. The activity of flavonoid and anthocyanin biosynthetic enzymes is primarily regulated transcriptionally by complexes that consist of various R2R3-MYBs, bHLH (basic helix–loop–helix), and WD40 (WD40-repeat-containing protein) (Grotewold, 2006; Sagawa et al., 2016).

The structural genes in the highly conserved carotenoid biosynthetic pathway have been well characterized in multiple plant model systems (Moehs et al., 2001; Ha et al., 2007; Chiou et al., 2010; Yamagishi et al., 2010; Yamamizo et al., 2010). In the carotenoid biosynthetic pathway, phytoene synthase enzyme (PSY) is considered a rate-limiting enzyme, and variations in its expression or activity alter the flux through the pathway (Yamagishi et al., 2010), and increased PSY abundance was achieved either through overexpression or by providing enhanced PSY stability increase in the total carotenoid content in plant tissues (Welsch et al., 2018). The carotenoid biosynthesis pathway starts from the C5 isoprene unit isopentenyl pyrophosphate (IPP) in the plastids. Four molecules of IPPs are condensed to form C20 geranylgeranylpyrophosphate (GGPP), which is catalyzed by IPP isomerase (IPI) and GGPP synthase (GGPS). Then, two GGPPs yield 15-cis phytoene by the action of phytoene synthase (PSY), which is converted into phytofluene, ζ-carotene, and lycopene by two desaturases: phytoene desaturase (PDS) and zeta-carotene desaturase (ZDS). All-trans-lycopene is synthesized by the action of carotenoid isomerase (CRTISO) and ζ-carotene isomerase (ZISO). Lycopene is cyclized to form α-carotene and β-carotene, which are catalyzed by lycopene β-cyclase (LCYB) and lycopene ε-cyclase (LCYE), respectively. α-Carotene and β-carotene are further modified by hydroxylation to convert xanthophyll by ε-hydroxylase (CHYE) and β-hydroxylase (CHYB), respectively. β-xanthophyll is epoxidated-de-epoxidated by zeaxanthin epoxidase (ZEP) to synthesize violaxanthin, which is converted to neoxanthin by neoxanthin synthase (NSY) (Moehs et al., 2001; Ha et al., 2007; Chiou et al., 2010; Yamagishi et al., 2010; Yamamizo et al., 2010).

Recently, several transcription factors families have been demonstrated to directly regulate the carotenogenic genes and control carotenoid biosynthesis in several model plants. In the tomato fruit, downregulation of *SlMYB72* altered the expression levels of genes involved in the biosynthesis of chlorophylls, carotenoids, and flavonoids (Wu et al., 2020). In *Arabidopsis*, the bHLH transcription factor PHYTOCHROME INTERACTING FACTOR1 (PIF1) and the bZIP TF LONG HYPOCOTYL5 (HY5) antagonistically regulate carotenoid accumulation by directly binding to the promoter of *PSY* in response to light stimulation (Toledo-Ortiz et al., 2014). *In Citrus calli*, overexpression of *CsMADS6* was associated with transcriptional activation of certain key genes involved in carotenoid biosynthesis (Lu et al., 2018). In *Nicotiana benthamiana*, overexpression of MYB7 stimulated the transcriptional activation of certain key genes involved in carotenoid biosynthesis (Ampomah-Dwamena et al., 2019). WHITE PETAL1 (WP1), an anthocyanin-related R2R3-MYB protein, played critical role in regulating floral carotenoid pigmentation in *Medicago truncatula* (Meng et al., 2019). Although the mechanisms of flower coloration in model plants have been well documented, the molecular mechanisms underlying flower coloration in *P. vulgaris* remain elusive. Transcriptional analysis of flower coloration via RNA sequencing (RNA-seq) has been successfully applied to many species, including *Muscari armeniacum*, *Paeonia rockii*, *Lilium* spp., and *Paeonia suffruticosa* Andr. (Lou et al., 2014; Zhang et al., 2015; Suzuki et al., 2016; Shi et al., 2017). Thus far, no RNA-seq studies of color variation in the flowers of *P. vulgaris* have been reported.

In the present study, the RNA-seq analysis of four cultivars of *P. vulgaris* with four different flower colors was conducted. Furthermore, by combining anatomical and biochemical analyses with bioinformatics, the molecular basis of the physiological processes involved in flower coloration in *P. vulgaris* was characterized. This work reveals how transcriptional profiles influence physiological and biochemical characteristics, resulting in flower coloration in *P. vulgaris*. Our results could lay a foundation for further studies of gene expression and functional genomics in primrose.

## Materials and Methods

### Plant Material

**FIGURE 1:**
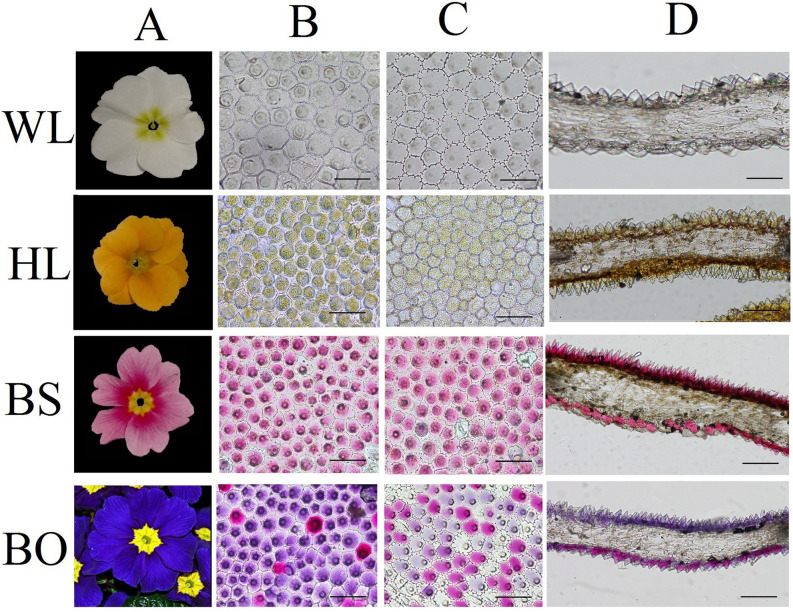
Cellular features of the flower materials. **(A)** Mature inflorescences of different cultivars; **(B)** epidermal cells of adaxial petals; **(C)** epidermal cells of abaxial petals; **(D)** cross- sections of petals. WL, HL, BS, and BO represent “White Lover,” “Huang Li,” “Beautiful Scenery,” and “Blue Onstar,” respectively. Bars, 100 μm.

The seeds of *P. vulgaris* cultivars with different flower colors were obtained from Shanghai Hemei Gardening Co., Ltd. (Shanghai, China) and planted in an experimental greenhouse at Northwest A&F University, Yangling, Shaanxi, China. The four *P. vulgaris* cultivars tested were “White Lover” (white), “Beautiful Scenery” (pink), “Huang Li” (yellow), and “Blue Onstar” (blue), abbreviated as WL, BS, HL, and BO, respectively (Figure 1). Fully opened and pigmented flowers with exposed anthers were harvested for further morphological observation and pigment content determination as well as RNA-seq. For morphological and anatomical observations, the petals were harvested and were used immediately for the observations and for the preparation of protoplasts. For pigment content determination and RNA extraction, tissues were snap-frozen in liquid nitrogen and stored at −80°C. The white cultivar “White Lover” was used as a control throughout the study.

### Measurement of Petal Color

Petal color was determined in the middle region of the fully pigmented flowers at noon, indoors, and with a north light using the Royal Horticultural Society Color Chart (RHSCC). The RHSCC remains a valuable tool for those evaluating plant colors and for those using published descriptions to visualize the colors. Fully pigmented flowers were randomly sampled and placed in a single layer on a six-well culture plate (Corning Costar, Sigma-Aldrich, Billerica, MA, United States). Their color parameters were measured with a Chroma meter (CR-410, Konica Minolta Sensing, Inc., Osaka, Japan) based on the CIE *L^∗^*a*^∗^*b*^∗^* scale (Zhang et al., 2007). All experiment analyses were based on five biological replicates of each sample and three technical replicates of each biological replicate.

### Scanning Electron Microscopy and Microscopic Observation

SEM was employed to study the petal epidermal surfaces of different *Primula* cultivars. The middle portions of the urn-shaped flowers were fixed, dehydrated, and dried according to previously described methods (Shi et al., 2017). The samples were mounted on a specimen stub and sputter-coated with gold before examination by scanning electron microscopy [JSM-6360 LV SEM, JEOL (Beijing) Co., Ltd., Beijing, China].

Fresh flowers were cross-sectioned. The adaxial and abaxial epidermal layers were peeled off using a razor blade. The epidermal layers were placed on a glass slide with a drop of water and then were immediately observed under a light microscope (BX43, Olympus, Tokyo, Japan) equipped with a DS cooled camera head with the FNIS-Elements image processing software.

### Analysis of Flavonols, Anthocyanins, and Carotenoids

The total concentration of flavonol, anthocyanin, and carotenoid were calculated based on a simple linear regression using malvidin-3,5-di-O-glucoside (Mv3G5G), rutin, and b-carotene as the standards at 520, 350, and 440 nm, respectively. The flavonol, anthocyanin, and carotenoid contents were calculated in milligrams per gram dry weight (as a quantity of Mv3G5G in mg/g, a quantity of rutin in mg/g, and a quantity of b-carotene in mg/g, respectively). The absorbance was detected by ultraviolet spectrophotometer (UV759S, Jingke, Shanghai, China).

A total of 10 mg of lyophilized petal powder from 10 individuals of different-colored *P. vulgaris* cultivars was extracted in 1 ml of 0.1% acetic acid/methanol solution at 4°C overnight and then centrifuged for 10 min at 10,000 rpm. The supernatant was collected and dried with a vacuum centrifuge concentrator (CV100-DNA, Aijimu, Beijing, China). The identification and quantification of flavonoid and anthocyanin compounds were performed with an ultra-high performance liquid chromatography–mass spectrometry coupled to a triple-Quadrupole Mass Spectrometry (XEVO^®^-TQ, Waters, Milford, MA, United States) with electrospray ionization (ESI). The separation was carried out with a ZORBAX Eclipse plus C18 (150 mm × 3.0 mm) with particle size of 1.8 μm (Agilent Technologies, United States) at 40°C. The gradient was as follows: 0.1% formic acid (A) and acetonitrile (B) were used as the mobile phases at 0–1 min (5% B), 1–8 min (5%–30% B), 8–12 min (40–95% B), 16–17 min (95–100% B), 17–21 min (100% B), and 21–25 min (5% B). The operating conditions were as follows: a flow rate of 1.0 ml/min^–1^ in positive ion ESI mode, capillary voltage at 3.0 kV and nitrogen flow at 16 L/h of nebulization. The compounds of the sample extract were identified by comparing the retention times, the characteristics of the UV-Vis spectra of peaks and the mass spectrometric information of the standard compounds. The relative quantification content was calculated from the peak areas of characteristic mass spectrometry (MS) daughter ion peaks based on the intensity of the corresponding standard compounds for flavonols (Harborne, 1968, 1969), including kaempferol, quercetin-3-glucoside-7-gentiobiosiden, quercetin, rutin, quercetin-3-glucoside, myricetin, kaempferol-3-sophoroside, kaempferol-3-rutinoside, quercetin-3-sophoroside; and for anthocyanins, including pelargonidin-3,5-diglucoside, delphinidin, pelargonidin, petunidin-3-glucoside, peonidin-3-glucoside, peonidin-3,5-diglucoside, peonidin-3-rutinoside, delphinidin-3-glucoside, cyanidin-3-rutinoside, and delphinidin-3,5-diglucoside. MassLynx^TM^ (V 4.1, SCN 846, Waters Corp., Manchester, United Kingdom) was used for MS data acquisition and data analysis. The chromagraphs were plotted using OriginPro 2015.

In the meantime, 30 mg of lyophilized petal powder from 10 individuals of different-colored *P. vulgaris* cultivars were extracted for carotenoid component analysis with acetone-hexane (1:2) three times for 30 min each and then were analyzed using ultra-high-performance supercritical fluid chromatography–mass spectrometry (UHPSFC-MS) techniques. The UHPSFC-MS analysis was carried out using a Waters Acquity UltraPerformance Convergence Chromatography (UPC^2^) system (Waters, Milford, MA, United States) coupled to a triple-Quadrupole Mass Spectrometer (XEVO^®^-TQ) with electrospray ionization (ESI). The column (HSS C18 SB column 100 mm × 3 mm, 1.8 μm) was purchased from Waters (Milford, MA, United States), and the separation of carotenoids was achieved using UHPSFE under the following conditions: mobile phase A, CO_2_; mobile phase B, 100% ethanol; flow rate, 1.5 ml/min; injection volume, 1 μl; column temperature, 40°C. The following gradient program was used: 0–0.5 min, 5% B; 0.5–5 min, 5–20% B; 5–7 min, 20% B; 7–8 min, 20–5% B; and 8–10 min, 5% B. The MS data acquisition was performed in positive mode using the ESI under the following conditions: data acquisition via UV-vis detection at 450 nm, MS in the range of m/z 100–800 Da, capillary voltage of 3 kV, cone voltage of 30 V, desolvation temperature of 300°C, and cone gas flow rate of 50 L/h. MassLynx^TM^ (V 4.1, SCN 846, Waters Corp., Manchester, United Kingdom) was used for MS data acquisition and data analysis. The chromagraphs were plotted using OriginPro 2015.

The compounds of sample extracts were identified by comparisons with the retention times of the standards, the characteristics of the UV-Vis spectra of the peaks and the MS information using Mass Hunter qualitative software. The relative quantification of the anthocyanin and flavonoid content was calculated from peak areas of the samples based on the intensity of the corresponding standard compounds, including lutein, β-carotene, lycopene, cryptoxanthin, and trans-β-Apo-8′-carotenal as internal standard for carotenoids. For compounds lacking corresponding standards, the quantification was carried out using similar compounds (Harborne, 1968, 1969). The correlation between the content of pigment content and the parameters of flower color was assessed by Pearson correlation coefficient using the statistical software IBM SPSS19.0 (IBM, Armonk, NY, United States) (Wang et al., 2018). All reactions—technical and biological—were performed in triplicate.

### cDNA Library Construction

Total RNA was extracted from 1 mg of pooled samples of petals from five different individuals of WL, HL, BS, and BO using TRIzol (Invitrogen, Carlsbad, CA, United States). Then, RNA quality was tested on an agarose gel and by a NanoDrop 8000 spectrophotometer (NanoDrop, Thermo Scientific, Waltham, MA, United States) and an Agilent 2100 Bioanalyzer (Agilent Technologies; Palo Alto, CA, United States). The cDNA libraries for the four different cultivars were constructed using an Illumina kit (Illumina, San Diego, CA, United States). Three biological replicates were conducted for each cultivar. The RNA-seq was performed using the Illumina HiSeq^TM^ 2500 platform at the Biomarker Biotechnology Corporation (Beijing, China). Then, the clean data were deposited in the National Center for Biotechnology Information (NCBI) Sequence Read Archive database under the accession no. SRP120574.

### Differential Gene Expression Analysis

The cleaned reads were assembled *de novo* using Trinity software (Grabherr et al., 2011). edgeR was used to measure the relative abundance of transcripts with the FPKM (fragments per kilobase per million mapped reads) method (Ali et al., 2008). The identification of significant digital gene expression (DGE) models between samples was performed with Cuffdiff (Trapnell et al., 2010). Differentially expressed transcripts (FDR value≤0.001 and ≥1.5-fold change) were annotated and categorized automatically with the Blast2GO GO and KEGG databases (Zhang and Wiemann, 2009; Zhang et al., 2015). To analyze the interactions among genes related to flower coloration, a network of all differentially expressed pigmentation-related genes and metabolites were constructed using the R package “weighted correlation network analysis” according to a previously published protocol (Langfelder and Horvath, 2008; Miller et al., 2010). The Pearson correlation coefficient was calculated in the R environment^1^ with its “base” function and “stat” packages. Two nodes were determined to be connected if the absolute value of Pearson’s correlation coefficient exceeded 0.93 (Zheng and Zhao, 2013). The coexpressed isoforms with strong interconnection were considered as hub isoforms. Then, the networks were visualized using the Cytoscape software (version 3.1.0).

### Quantitative Real-Time PCR Verification

To estimate the validity of the transcriptome sequencing data, 30 genes associated with flower coloration were selected randomly and analyzed with qRT-PCR. Total RNA was extracted using RNA Extraction kit (Tiangen, China) and digested by DNase I (Takara, Japan). An aliquot of 2 μg of total RNA was reverse-transcribed to first-strand cDNA using M-MLV (Promega, Madison, WI, United States). The primers were designed using Oligo dT7 and the Primer 3 software (Supplementary Table S10). qRT-PCR was conducted according to previously described methods (Shi et al., 2017). The relative expression levels of genes in the petals were normalized to the *actin* (c55882) gene expression level in the same sample and were calibrated to the transcript levels in the WL petals. All reactions were performed in triplicate both technically and biologically.

### Protoplast Preparation, Transfection, and Transient Expression Analysis

The preparation of free protoplasts from adaxial and abaxial epidermal layers was performed according to previously described methods (Yoshida et al., 2009a, b; Qi et al., 2013). The released protoplasts were observed under a microscope.

The subcellular localization and overexpression analysis of three MYBs were performed by transfecting GFP-tagged MYB into protoplasts of WL petals. The full-length cDNA of c44135 (MYB-1), c40864 (MYB-2), and c39130 (MYB-3) were fused in frame with the GFP cDNA and ligated between the CaMV 35S promoter and the nopaline synthase terminator, respectively. The primers used for complete open reading frame (ORF) cloning of MYB-1, MYB-2, and MYB-3 are shown in Supplementary Table S11. WL protoplasts were collected by horizontal centrifugation and resuspended in MMG solution (15 mM MgCl_2_, 0.6 M mannitol, 10 mM MES, pH 5.7) to reach a concentration of 2 × 10^6^ cells/ml. For transfection experiment, 1 ml protoplasts were transfected with 10 μg plasmids. As a control, cotransfection of empty plasmid of GFP was carried out. The transfected cells were incubated at 25°C in the dark for 18 h and used for further analysis. The fluorescence signals in transfected protoplasts were examined using a confocal laser scanning microscope (Leica Microsystems, Japan). The protoplast transformation efficiency was 74%. Total RNA extracted from WL protoplasts was digested by DNase I (Takara, Japan) to get rid of the transfected plasmids and synthesized to be cDNA as above. The expression pattern of putative MYB-regulated genes was conducted by qRT-PCR according to previously described methods (Shi et al., 2017). All experiments were performed in triplicate both technically and biologically.

## Results

### Anatomical Variation in Differentially Colored Flowers

To study the effects of the petal structure on flower coloration, the spatial locations of pigments were examined in the petals of the four *P. vulgaris* cultivars. The cell shapes on both epidermal surfaces for all cultivars were found to be multipapillate under optical microscope, except for the abaxial epidermis of “Beautiful Scenery” (BS) with a pink flower color and “Huang Li” (HL) with a yellow flower color, where the cell shapes appeared rounded (Figures 1B,C). In the blue flower “Blue Onstar” (BO), the colored cells exhibited a variety of colors on both epidermal surfaces, ranging from amaranth to blue. In addition, the adaxial epidermis of BO showed much deeper coloration than the abaxial epidermis. Interestingly, the adaxial epidermis of BO mainly contained blue cells, while the abaxial one was populated by amaranth cells (Figures 1B,C). Furthermore, the flower color of BO did not arise from a single type of protoplast but from a mixture of amaranth purple and blue protoplasts (Supplementary Figure S1). This result agreed with the observations from transverse sectioning.

**FIGURE 2:**
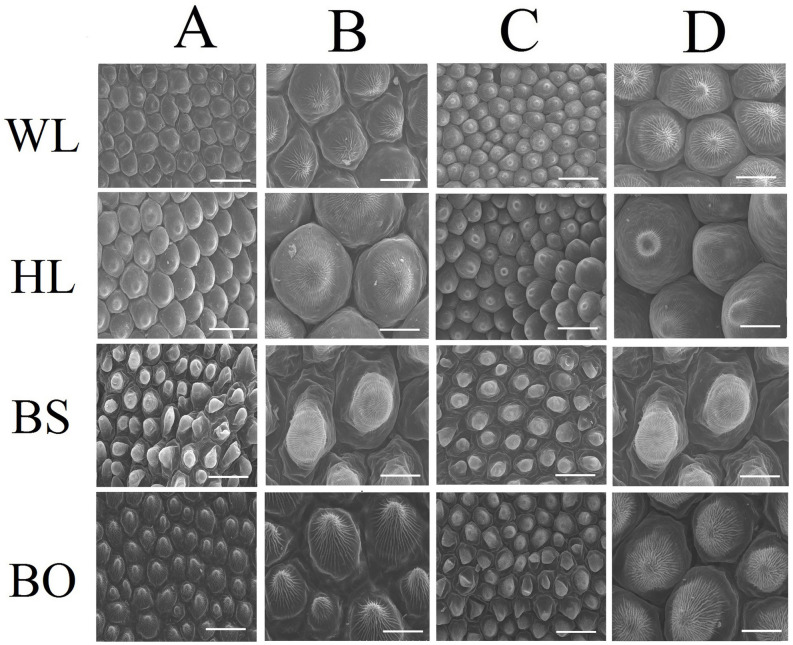
Scanning electron microscopy (SEM) images of papillate cells from the adaxial epidermis **(A,B)** and abaxial epidermis **(C,D)** of petals. WL, HL, BS, and BO represent “White Lover,” “Huang Li,” “Beautiful Scenery,” and “Blue Onstar,” respectively. Bars, 40 μm **(A,C)** and 20 μm **(B,D)**.

Observations based on scanning electron microscopy (SEM) indicated that papillae were pronounced and present on the petal surfaces of all four cultivars. The papillae on the adaxial surface of BO appeared triangular, and those on BS appeared sword-like (Figure 2). In contrast, the papillae on the adaxial surfaces of WL and HL were much smoother and appeared spherical, as did the papillae on the abaxial epidermis of WL, BS, and HL. Generally, the papillae of WL and HL were more crowded and rounder than those of BS and BO (Figure 2).

### Color Index Measurement

**FIGURE 3:**
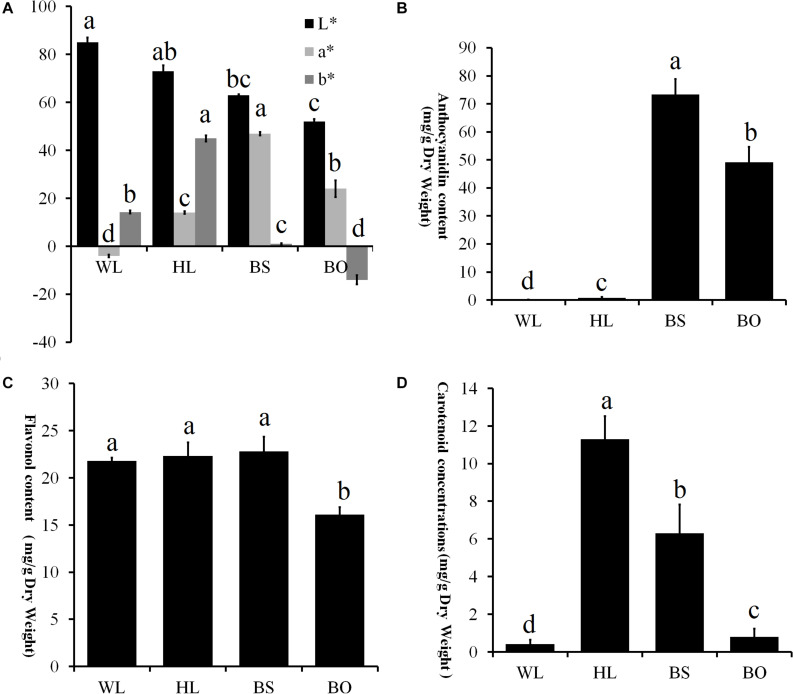
Statistics of petal color parameters and total content of anthocyanin, flavonoids, and carotenoids. **(A)** Petal color parameters of the four different cultivars. L* lightness; a*, b* chromatic components. A high L* value indicates a lighter color. The parameter a* indicates the balance between red and green, and the parameter b* indicates the balance between yellow and blue. **(B)** Total content of anthocyanin (mg/g) in the fully open petals of four different cultivars. **(C)** Total content of flavonoids (mg/g) in the fully open petals of four different cultivars. **(D)** Total content of carotenoids (mg/g) in the fully opened petals of four different cultivars. WL, HL, BS, and BO represent “White Lover,” “Huang Li,” “Beautiful Scenery,” and “Blue Onstar,” respectively. All experiment analyses were based on five biological replicates of each sample and three technical replicates of each biological replicate. Different letters indicate significant difference (*P* < 0.05).

To comprehensively understand the genetic backgrounds of the different *P. vulgaris* cultivars, the color indices were measured. First, the flower colors were defined according to the Royal Horticultural Society Color Chart (RHSCC). The color grades of WL, HL, BS, and BO were 155B, 15B, 73B, and N95C, respectively. Moreover, the flower color at the fully open stage was measured using a Chroma meter and was recorded as a three-dimensional International Commission on Illumination (CIE) *L^∗^a^∗^b^∗^* value. High *L*^∗^ values for WL and HL flowers were consistent with their brighter colors, while the lowest *L*^∗^ value of BO indicated a darker color (Figure 3A). The parameter *a*^∗^ had positive values in BS, HL, and BO but not in WL (Figure 3A). In BS, the highest *a*^∗^ value indicated a reddish color (Figure 3A). The parameter *b*^∗^ had a negative value in BO and the highest value in HL describing the blue color of BO and the yellow color of HL, respectively (Figure 3A).

### Pigment Variation in Differentially Colored Flowers

The pigments in the petals of the four cultivars were identified and quantified. In total, 15 anthocyanin, 20 flavonols, and 14 carotenoids were identified (Supplementary Tables S1–S3 and Supplementary Figures S2–S4). In an analysis of the total contents of color-determining compounds, anthocyanin content was found to accumulate at significantly higher levels in BS and BO, while anthocyanins were present in trace amounts in HL petals and entirely absent in WL petals (Figure 3B). The flavonol level of BO was significantly lower than the other three cultivars (Figure 3C). The carotenoid content was highest in HL, followed in order by BO and BS, and WL presented the lowest value (Figure 3D).

For the 15 anthocyanins identified in the petals of the four *P. vulgaris* cultivars (Supplementary Table S1 and Supplementary Figure S2), blue flowers contained six main anthocyanin compounds: delphinidin-, petunidin-, peonidin-, and malvidin-type anthocyanins; rosinin; and hirsutin, of which rosinin and hirsutin were previously found to be peculiar to *Primula* (Harborne, 1968). Hirsutin, hirstidin-4′-malonate, petunidin 7-methoxy-3,5-di*-O*-glucoside, malvidin 3-*O*-glucoside, and rosinin were most abundant in BO. In addition, pink flowers mainly accumulated peonidin-type anthocyanins, cyanidin 3-*O*-glucoside, and delphininidin 3,5- di*-O*-glucoside-3′-caffeic ester. Moreover, a small amount of pelargonidin 3,5-di*-O*-glucoside was also detected in the BO and BS petals.

Among 20 flavonols identified in the petals of the four cultivars (Supplementary Table S2 and Supplementary Figure S3), quercetin 3-gentiobioside-5-*O*-glucoside and kaempferol 3-*O*-gentiotrioside were more abundantly accumulated in BO, while quercetin 3-*O*-glucoside, quercetin 3,5-di*-O*-glucoside, kaempferol 3,5-di*-O*-glucoside, and isorhamnetin 3-*O*-glucoside-caffeic ester were mainly synthesized in BS. For the HL and WL petals, quercetin 3-gentiobioside-7-*O*-glucoside, and all of the identified gossypetin derivatives, including gossypetin 3,5-di*-O*-glucoside-8-caffeic ester, gossypetin 7-methoxy-3,5-di*-O*-glucoside-8-caffeic ester, gossypetin 7-methoxy-3,5-di*-O*-glucoside-8-caffeic ester, gossypetin 7,3*′*-dimethoxy-3,5-di*- O*-glucoside-8-caffeic ester, and gossypetin 7-methoxy-3-*O*-glucoside were more abundant in yellow flowers, while in the WL petals, quercetin and kaempferol flavonols were most abundant.

Additionally, 14 different profiles of carotenoid accumulation were identified in the four cultivars (Supplementary Table S3 and Supplementary Figure S4). The petals of HL and BS accumulated more abundant carotenoids with a high accumulation of antheraxanthin dimethyl ester. The levels of acetyl-zeaxanthin, β-carotene, lutein methyl ester, cryptoxanthin methyl ester, and (all-E)-violaxanthin/(9Z)-violaxanthin were also high. In contrast, almost no detectable carotenoids could be found in the petals of BO and WL.

Furthermore, the correlation analysis between the content of each component derivatives and the color index (*L^∗^a^∗^b^∗^*) showed that herbacetin exhibited the highest correlation coefficient with *L*^∗^ (0.9951), peonidin- and cyanidin-type anthocyanins with *a*^∗^ (0.9159 and 0.9099, respectively). There was a great correlation between gossypetin derivatives and *b*^∗^ with the correlation coefficient 0.9219 (Supplementary Table S4).

### KEGG Pathway Analysis

To understand the molecular basis of flower coloration in *P. vulgaris*, petals of the four cultivars were used to construct 12 libraries for high-throughput sequencing. Among all 101,112 unigenes, a total of 46,829 unigenes were annotated based on public databases (Supplementary Table S5). Compared with WL, 381 differentially expressed genes (DEGs) were simultaneously upregulated, and 325 DEGs were simultaneously downregulated in the HL, BO, and BS cultivars (Supplementary Figure S5).

**FIGURE 4:**
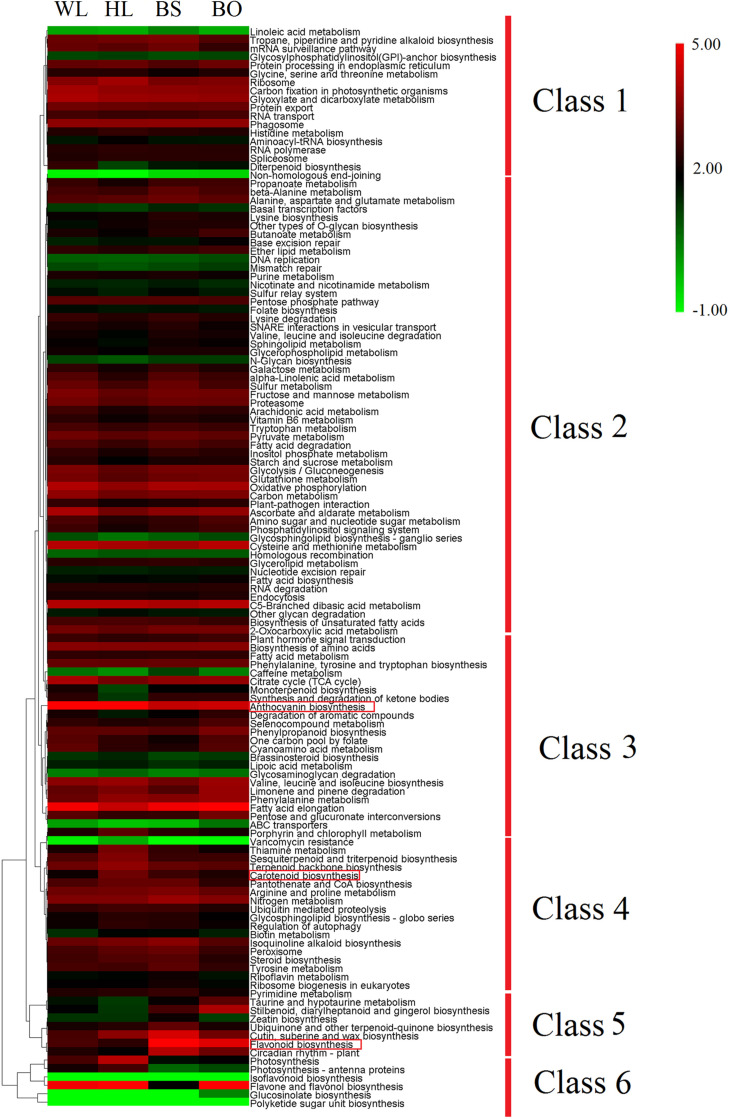
Expression profiles of 127 pathways in KEGG (Kyoto Encyclopedia of Genes and Genomes). The heatmap was generated by the average expression level of the genes in each pathway based on log ratio fragments per kilobase per million (FPKM) data. The color scale represents log_2_ transformed FPKM values. Green indicates low expression, and red indicates high expression. WL, HL, BS, and BO represent “White Lover,” “Huang Li,” “Beautiful Scenery,” and “Blue Onstar,” respectively.

To obtain further insights into the different transcriptomic landscapes among different-colored flowers, we performed a Kyoto Encyclopedia of Genes and Genomes (KEGG) pathway enrichment analysis. The corrected *p*-value (*q*-value) of the richness factor was used to evaluate the importance of the pathways in each cultivar. For WL vs. HL, the genes involved in “anthocyanin biosynthesis,” “carotenoid biosynthesis,” and “phenylpropanoid biosynthesis” were the most significantly enriched (Supplementary Figure S6A). For WL vs. BS, genes involved in “flavonoid biosynthesis,” “anthocyanin biosynthesis,” “flavone and flavonol biosynthesis,” and “carotenoid biosynthesis” were the most significantly enriched (Supplementary Figure S6B). For WL vsvs. BO, “glucosinolate biosynthesis,” “flavonoid biosynthesis,” “phenylpropanoid biosynthesis,” “anthocyanin biosynthesis,” and “flavone and flavonol biosynthesis” were the most significantly enriched (Supplementary Figure S6C). To understand the overall expression trends of genes in each KEGG pathway, we calculated the average expression level of genes. The expression levels of “flavonoid biosynthesis”-related genes in BS and BO were nearly four times higher than those in WL, while those of the genes in “carotenoid biosynthesis” (Figure 4) in HL and BS were about two times higher than those in WL. The expression of “anthocyanin biosynthesis” genes showed the highest expression levels in HL. Our KEGG pathway analysis confirmed that anthocyanin-, flavonoid-, and carotenoid-associated genes play important roles in *P. vulgaris* flower coloration. To validate the DEGs identified by RNA-seq, quantitative reverse transcription PCR (qRT-PCR) was performed using the fully opened petals of the four cultivars. As expected, the qRT-PCR data and RNA-seq data were in close agreement based on Pearson’s correlation coefficient (*R*^2^ = 0.8453, *p* < 0.0001), indicating that the transcriptome results were highly reliable (Supplementary Figures S7, S8).

### Expression Analysis of Flavonoid Biosynthetic Genes

**FIGURE 5:**
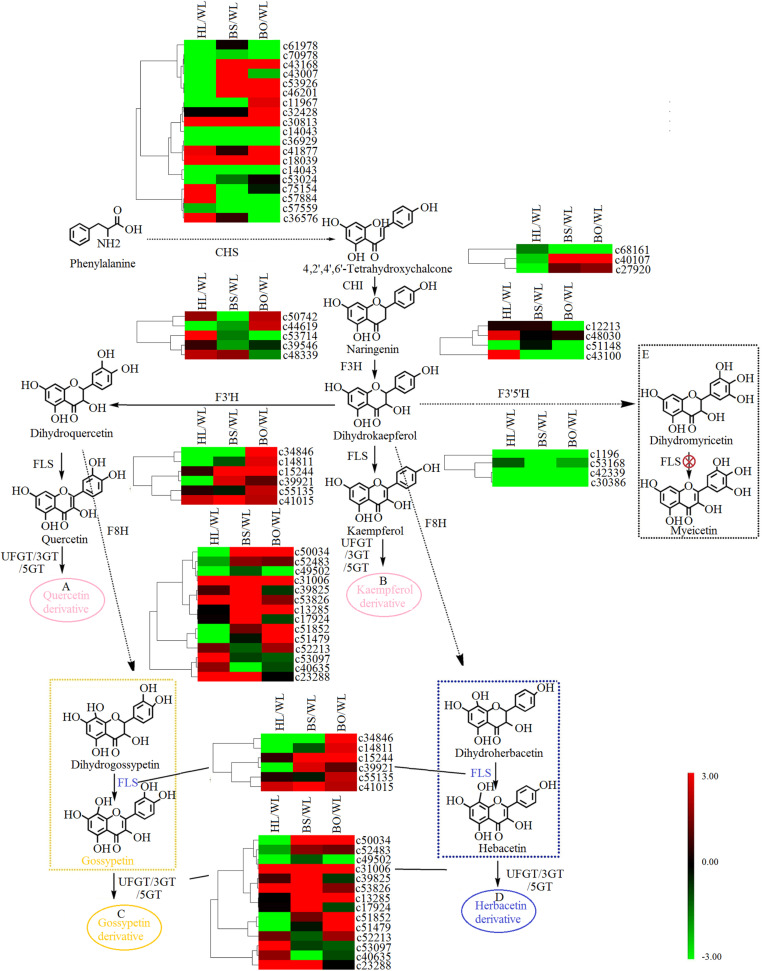
A detailed schematic of the flavonoid biosynthetic pathway as related to flower pigmentation in the four different cultivars of *Primula vulgaris*. Enzyme names and expression patterns are listed at each step. The expression level of each gene in each sample was averaged from three biological replicates of transcriptome sequencing. The color scale represents log2-transformed fold change values. Black indicates no change, green indicates downregulation, and red indicates upregulation. WL, HL, BS, and BO represent “White Lover,” “Huang Li,” “Beautiful Scenery,” and “Blue Onstar,” respectively. **(A)** In pink ellipse: quercetin-related compounds; **(B)** in pink ellipse: kaempferol-related compounds; **(C)** in yellow ellipse: gossypetin-related compounds; **(D)** in blue ellipse: herbacetin-related compounds. The yellow dashed frame represents the key steps of flavonoid synthesis in the yellow-flowered cultivar; the blue dashed frame represents the key steps of flavonoid synthesis in the blue-flowered cultivar; and the black dashed frame represents a lack of flavonoid synthesis in *P. vulgaris* cultivars.

Based on the annotation results, a total of 48 unigenes were associated with flavonoid biosynthesis (Supplementary Tables S6, S7), including 27 DEGs. CHS is the first key enzyme of flavonoid biosynthesis, and most *CHS* members showed no significant expression difference in four varieties (Figure 5). Two *CHI* genes [*c40107* (type II) and *c27920* (type IV)] showed significantly increased expression in BS and BO. A previous study indicated that types I and II proteins are having CHI enzymatic activity, while types III and IV are not (Morita et al., 2014). Thus, *c40107* played critical roles in flavonoid biosynthesis. The gene *c43100* (F3H) was expressed most highly in HL and could be related to the biosynthesis of dihydrogossypetin. The *F3Th*, *FLS*, and *F3nd o*genes played key roles in different flavonol biosynthetic branches that could underlie various flower colors. *c53714* (*F3′H*) exhibited the most significantly increased expression in HL. Among the *FLS* unigenes, *c34846* was highly expressed in WL and BO, *c15244* was highly expressed in BS and BO, and *c14811* was upregulated in BO. *F3n BO* showed a high accumulation level in WL. Among the 3-*O*-glucosyltransferase (3GT) genes, *c50034* and *c53826* had the highest expression level in BS, and *c51852* and *c51479* were highly expressed in BO. *c39825* and *c53907* were highly expressed in BS and HL, respectively (Figure 5).

### Expression Analysis of Anthocyanin Biosynthetic Genes

**FIGURE 6:**
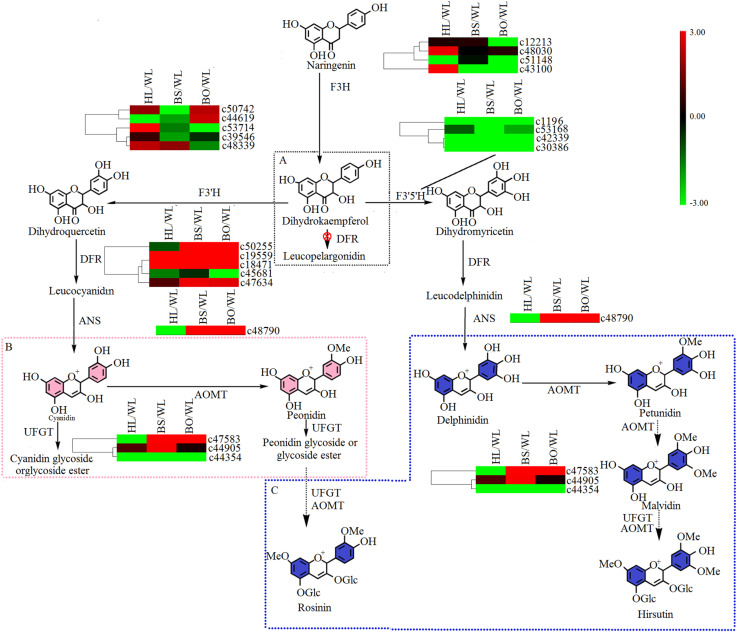
A detailed schematic of the anthocyanin biosynthetic pathway as related to flower pigmentation in the four different cultivars of *P. vulgaris*. Enzyme names and expression patterns are listed at each step. The expression level of each gene in each sample was averaged from three biological replicates of transcriptome sequencing. The color scale represents log2-transformed fold change values. Black indicates no change, green indicates downregulation, and red indicates upregulation. WL, HL, BS, and BO represent “White Lover,” “Huang Li,” “Beautiful Scenery,” and “Blue Onstar,” respectively. **(A)** The black dashed frame represents a lack of anthocyanin synthesis in *Primula vulgaris* cultivars; **(B)** the pink dashed frame represents the key step of anthocyanin synthesis in the pink-flowered cultivar; and **(C)** the blue dashed frame represents the key step of anthocyanin synthesis in the blue-flowered cultivar.

There were two main branches from dihydrokaempferol into different anthocyanins (Supplementary Tables S6, S7 and Figure 6). For BS and BO, cyanidin and delphinidin were biosynthesized from dihydroquercetin and dihydromyricetin and catalyzed by DFR and ANS, respectively. The expression levels of *DFR* (*c50255*) and *ANS* (*c48790*) were significantly increased in both BS and BO. Later, peonidin-based anthocyanins, which contribute to pink flower colors, were converted from cyanidin through a series of glycosylation and methylation reactions. Among the *anthocyanin O*-*methyltransferase* (*AOMT*) genes, *c47583* showed a high accumulation level in BO, followed by BS, although it was nearly undetectable in HL and WL. Peonidin-based anthocyanins were also methylated at the 7-*O* position and glycosylated at the 3- and 5-*O* positions to form rosinin, which was a unique anthocyanin found in BO. In BO, hirsutin, malvidin, and petunidin were formed from delphinidin by a series of methylation and glycosylation reactions to produce blue petals.

### Expression Analysis of Carotenoid Biosynthetic Genes

**FIGURE 7:**
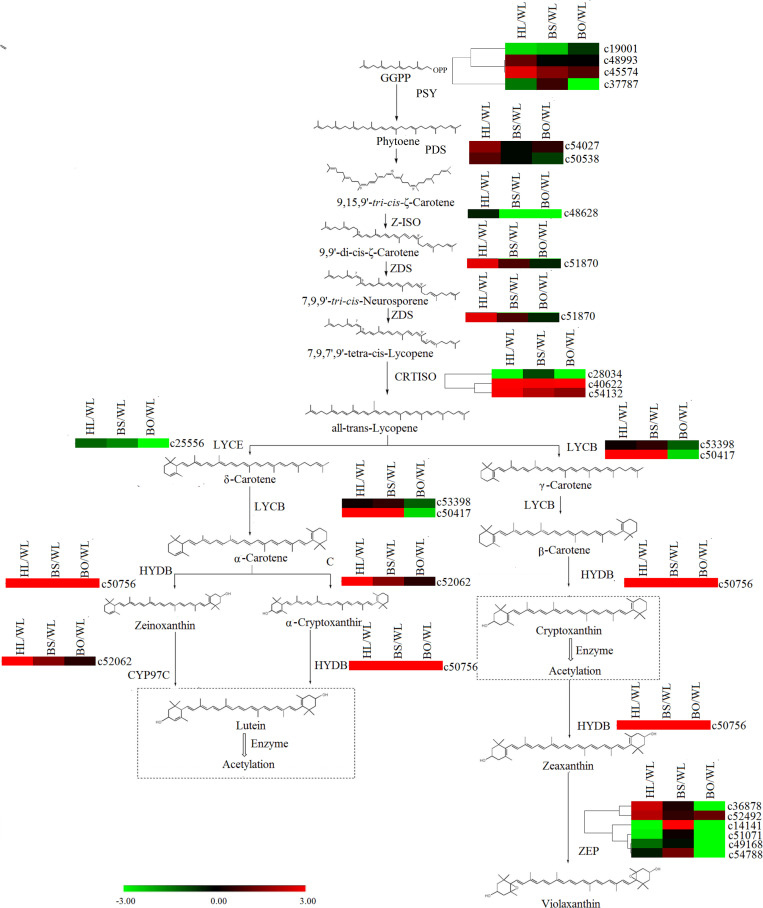
A detailed schematic of the carotenoid biosynthetic pathway as related to flower pigmentation in the four different cultivars of *P. vulgaris*. Enzyme names and expression patterns are listed at each step. The expression level of each gene in each sample was averaged from three biological replicates of transcriptome sequencing. The color scale represents log2-transformed fold change values. Black indicates no change, green indicates downregulation, and red indicates upregulation. WL, HL, BS, and BO represent “White Lover,” “Huang Li,” “Beautiful Scenery,” and “Blue Onstar,” respectively.

In the carotenoid biosynthetic pathway, all of the unigenes encoding *phytoene synthase (PSY)*, *phytoene desaturase (PDS)*, *ε-carotene isomerase (Z-ISO)*, and *ε-carotene desaturase (ZDS)* were significantly upregulated in HL (Supplementary Tables S6, S7 and Figure 7), which resulted in sufficient substrates for the downstream products. Among the *carotenoid isomerase (CRTISO*) genes, *c53132* was expressed at high levels in HL and BS. In line with the greater abundance of β-carotene than α-carotene in HL and BS, c50417 (*lycopene β-cyclase*, *LYCB*) showed relatively high transcription levels in HL and BS. *c52062* (*carotene ε-ring hydroxylase*, *CYP97C*) and *c50756 (β-carotene hydroxylase*, *HYDB)* exhibited the same profiles as the cryptoxanthin- and zeaxanthin-type derivatives in all four cultivars. However, none of the unigenes encoding *zeaxanthin epoxidase* (*ZEP*) showed accordant expression levels.

### Transcriptional Regulation of Flower Coloration

Among 92 unigenes annotated as *R2R3*-*MYB* TFs, 35 unigenes were identified as DEGs (Supplementary Tables S8, S9). c38443 was expressed relatively highly in HL and BS, while *c50767* and *c40864* showed higher expression levels in BS and BO. The expression level of *c48106* in HL was the highest, and *c36030* was most abundant in BO. However, *c19192* was downregulated in HL and BO with relatively low FPKM values. With regard to *bHLH* TFs, 33 unigenes were identified as DEGs (Supplementary Table S9). *c19199* was highly expressed in HL and BS, and *c46449* and *c50718* showed significantly increased expression in both WL and BO. *c36176* was significantly more abundant in BS. *c50758*, *c49536*, and *c18795* exhibited higher FPKM values in HL, while *c13543* and *c45751* were more abundant in BO. In addition, the identified *WD40* showed no significantly different expression levels among the four cultivars except that *c35345* was expressed at the highest level in BS, and *c54879* was highest in HL.

### Coexpression Network Analysis

**FIGURE 8:**
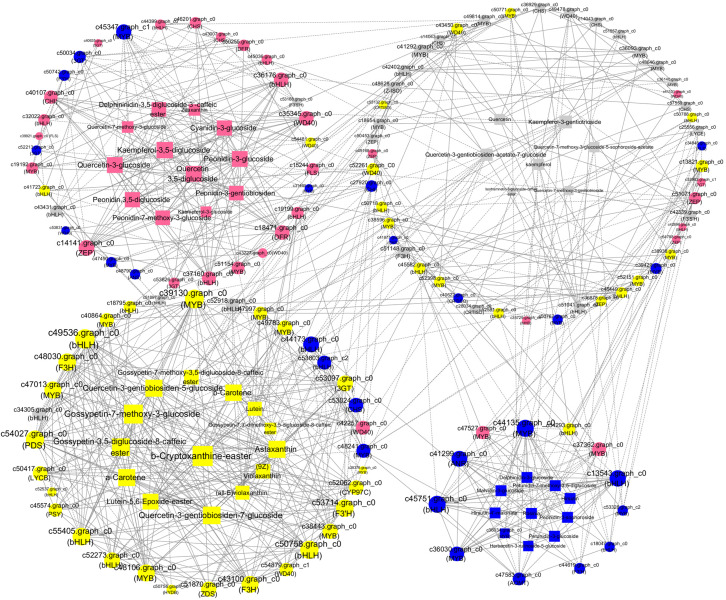
A coexpression network of the genes and metabolites involved in pigmentation and anthocyanin, flavonoid, and carotenoid accumulation. Silvery white, yellow, pink, and blue circles represented genes with the highest accumulation level in “White Lover,” “Huang Li,” “Beautiful Scenery,” and “Blue Onstar,” respectively. Silvery white, yellow, pink, and blue rectangle represented metabolites with the highest accumulation level in “White Lover,” “Huang Li,” “Beautiful Scenery,” and “Blue Onstar,” respectively. Larger circles or rectangles in the network indicate gene or metabolite edges with more connections. Edges or connections represent coexpression between isoforms with a Pearson correlation coefficient (PCC) < −0.95 (solid line with a T-type arrow) or ≥+0.95 (solid line with a normal arrow).

To analyze the interactions among anthocyanin biosynthesis, flavonoid biosynthesis, and carotenoid biosynthesis, PCCs (Pearson correlation coefficients) were calculated (Figure 8). The maximum numbers of correlations identified were found in *bHLH* (*c49536*), which shared positive correlations with one each of the *WD40*, *PDS*, *3GT*, *CYP97C*, *ZDS*, and *PSY* unigenes and with two *F3H*, three *MYB*, and three *bHLH* unigenes (Figure 8). In addition, the expression of *bHLH (c49536)* was positively correlated with the accumulation of quercetin-3-gentiobiosiden-7-glucoside, quercetin-3-gentiobiosiden-5-glucoside, gossypetin-7-methoxy-3-glucoside, gossypetin-3,5-diglucoside-8-caffeicester, antheraxanthin, and α-carotene. *ZEP* (*c14141*), *WD40* (*c35345*), *DFR* (*c18471*), and *bHLH* (*c36176*) as the hub genes showed high expression levels in BS, indicating that they might play important roles in pink coloration. The top hub genes included two *bHLH* (c*45751* and *c13543*) and one *MYB* (*c44135*) genes in the BO network. The *c44135* gene was positively correlated with *ANR* (*c41299*), *AOMT* (*c47583*), and many *MYB* and *bHLH* genes but negatively correlated with one *bHLH* (*c54293*) and one *MYB* (*c47527*) (Figure 8). The carotenoid biosynthesis genes were clustered in HL with some flavonoid biosynthesis genes: *CHS*, *MYB*, *bHLH*, and *WD40* members. In WL, two *bHLH* unigenes (*c52918* and *c34305*) were positively correlated with each other, suggesting that these genes participated in similar regulatory processes. These findings suggest a strongly connected and coregulated pathway, consistent with what is already known about the relationships among these genes in other plants, including tomato and rice (Ozaki et al., 2010; Jeremy et al., 2015). The coexpression network suggested that the MYB, bHLH, and functional many genes played important roles in anthocyanin, flavonoid, and carotenoid biosynthesis. Previous studies indicated that flavonoids and anthocyanin biosynthesis is determined by a combination of R2R3-MYB, basic helix–loop–helix (bHLH), and WD40-type transcriptional factors and their interaction; however, MYB TFs are widely reported to play a determinant role in flavonoids and anthocyanin coloration in various dicotyledonous plants (Xu et al., 2015; Zhou et al., 2016). Thus, we selected MYBs for further experimental verification.

In order to obtain the accurate function of the hub MYBs in a coexpression network, we used these MYBs as cue blast against TAIR database, and many previous published works that claimed MYBs participated in flavonoids, anthocyanins, or carotenoids biosynthesis (Mehrtens et al., 2005; Stracke et al., 2010; Ampomah-Dwamena et al., 2019; Meng et al., 2019; Wu et al., 2020), and three MYBs including c44135 (MYB-1), c40864 (MYB-2), and c39130 (MYB-3), which showed the highest expression level in BO, HL, and WL were closest match with these well-documented MYBs. Phylogenetic analysis indicated that MYB-1 belongs to the clade S7 including AtMYB1, AtMYB11, AtMYB12, and AtMYB111, which regulate the production of phenylpropanoids and flavonol (Mehrtens et al., 2005; Stracke et al., 2010; Peng et al., 2016). MYB-2 was clustered with AtMYB123 (S7 clade) that acts as a key determinant in the proanthocyanidin accumulation of developing seed (Ampomah-Dwamena et al., 2019; Meng et al., 2019; Wu et al., 2020). MYB3 was clustered with AtMYB108, AtMYB78 in clade S20, which participated in stress treatment and anther development (Christian et al., 2010; Peng et al., 2016). Thus, we speculated that these three MYBs might participate in *P. vulgaris* floral color pigmentation.

**FIGURE 9:**
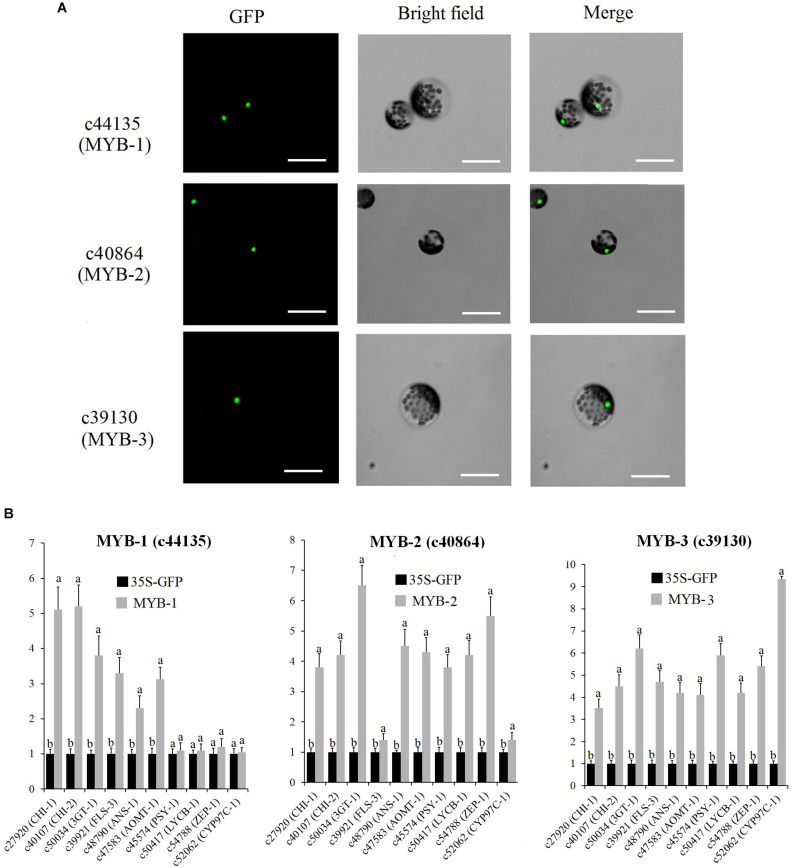
**(A)** Nuclear localization of c44135 (MYB-1), c40864 (MYB-2), and c39130 (MYB-3) in protoplasts of “White Lover” petals. Bars, 30 μm. **(B)** Transient overexpression of c44135 (MYB-1), c40864 (MYB-2), and c39130 (MYB-3) affected gene expression of flavonoid, anthocyanin, and carotenoid biosynthetic pathway. The qRT-PCR experiments were performed in triplicate both technical and biological. Different letters indicate significant difference (*P* < 0.05).

Subcellular localization analysis indicated that MYB-1, MYB-2, and MYB-3 were localized in the nuclei of protoplasts (Figure 9A). To determine the mechanism by which MYB-1, MYB-2, and MYB-3 regulate floral color pigmentation in *P. vulgaris*, we analyzed the transcript levels of some DEGs that positively correlated with accumulation of metabolites via protoplast transformation experiment. In order to ensure the reliability of protoplast transformation test, we compared the expression level of GFP label among different protoplast transformation tests. The qRT-PCR results showed a similar expression level of GFP in each protoplast transformation experiment (Supplementary Figure S10), indicating the stabile protoplast transformation efficiency in each overexpression test. In MYB-1 overexpression samples, most anthocyanin and flavonoid biosynthesis-related genes exhibited strong expression levels. However, no significant expression difference was detected by carotenoid-related genes such as ZEP-1. In terms of MYB-2, most anthocyanin, flavonoid, and carotenoid biosynthesis-related genes showed high accumulation levels in transfected protoplasts of MYB-2 (Figure 9B). In MYB-3 transgenic protoplast, OE-MYB-3 significantly increased the expression of anthocyanin, flavonoid, and carotenoid biosynthesis-related genes.

## Discussion

### The Crucial Role of Anthocyanins in Pink and Blue Flower Color Formation in *P. vulgaris*

Flower color depends on the contents of the colored cells (Quintana et al., 2007). Here, the adaxial petal surface in the BO individual visually appeared to be bluer than the abaxial surface and had a high proportion of blue cells, while the abaxial surface was mainly populated with amaranth cells (Figure 1 and Supplementary Figure S1). Meanwhile, in the WL, HL, and BS individuals, most cells were similar with no significant differences between the colors of the adaxial and abaxial epidermal cells. As expected, only a trace amount of anthocyanin was found in WL and HL flowers in contrast to the significantly abundant anthocyanins in BS and BO flowers. It was found that a blue color was due to hirsutin and rosinin in addition to petunidin- and malvidin-type anthocyanins. The peonidin-, cyanidin-, and delphinidin-type anthocyanins showed much higher accumulation levels in BS than in BO (Supplementary Table S1). These findings were consistent with the previous studies in Primulaceae (Harborne, 1968; Freyre and Griesbach, 2004; Quintana et al., 2007). Hirsutin, a 7-*O*-methylated anthocyanin, is confined to the genus *Primula* and is sometimes accompanied by a small amount of malvin and petunin through conversion by the AOMT and UFGT enzymes, whereas rosinin, a pigment related to hirsutin in the cyanidin series via *AOMT* and *UFGT*, is also rare (Harborne, 1968). Interestingly, these two rare pigments were detected only in the blue-flowered cultivar (Supplementary Table S1), which was consistent with previous studies (Harborne, 1968; Saleh Nabiel et al., 1988). Therefore, *AOMT* (c47583) was considered to function in BO.

In the anthocyanin biosynthetic pathway, cyanidin and delphinidin derivatives represent two independent branches from dihydrokaempferol, which are catalyzed by *F3′H* and F3′5′H, respectively. However, the differences in the anthocyanin compositions of BS and BO were consistent with the expression profiles of *F3′H* (*c53714*) and *F3′5′H* (*c53168*) in BO and BS (Figure 6 and Supplementary Table S7). Peonidin was the most abundant in BS petals (Supplementary Table S1), being synthesized from cyanidin via catalysis by AOMT. Additionally, we speculated that the AOMT enzyme catalyzing the synthesis of peonidin was *c44905*, which was different from its activity in BO, in which it catalyzed the methylation of delphinidin, malvin, petunin, and peonidin.

Studies have shown that UFGT is the key enzyme for anthocyanin biosynthesis in the berry skins of grape and litchi, and its expression is closely associated with anthocyanin concentrations (Zhao et al., 2012). In the flavonoid biosynthetic pathway, synthesized flavonols were further modified through a series of glycosylation steps catalyzed by 3GT to form stable derivatives. Thus, *c50034*, which showed high expression levels in BS and BO (Figure 5 and Supplementary Table S7), is believed to modify the anthocyanidins in BS and PO petals. In plants, flavones and flavonols as copigments influence the flower colors produced by anthocyanins, e.g., copigmentation effects render flowers bluer in *Torenia fournieri* (Aida et al., 2000). Among the four cultivars, herbacetin and kaempferol were more abundant in BO petals (Supplementary Table S2). In short, the blue color was due to hirsutin, rosinin, petunidin, and malvidin with the copigment herbacetin, while peonidin and delphinidin were responsible for the pink color under the control of different anthocyanin biosynthetic genes such as *F3′H* (*c53714*), *F3′5′H* (*c53168*), *AOMT* (*c47583*, *c44905*) and *3GT* (*c50034*).

### The Potential Factors Involved in Yellow Flower Coloration

In many plants, yellow flavonols accompanying yellow carotenoids contributed to the yellow flower coloration. Quercetagetin and gossypetin appeared to be important yellow pigments, particularly as carotenoids had not been identified in *P. vulgaris* at that time (Harborne, 1965, 1968). In the present work, quercetin 3-gentiobioside-7-*O*-glucoside and yellow flavonol glycosides based on gossypetin were also significant components in the HL cultivar, which partly agrees with previous reports (Harborne, 1965, 1968). In the flavonoid biosynthetic pathway, *F3H* (c43100) and *F3′H* (*c53714*), playing key roles in flavonol biosynthetic branches, exhibited the most significantly increased expression in HL. Thus, they could be related to the biosynthesis of quercetin and gossypetin. Moreover, in the coexpression network, the *c53097* (*3GT*) as UFGT members, which expressed most highly in HL petals, were closely connected with the accumulation level of quercetin 3-gentiobiosiden-7-*O*-glucoside, quercetin 3-gentiobiosiden-5-*O*-glucoside, gossypetin 7-methoxy-3-*O*-glucoside, gossypetin 3,5-di-*O*-glucoside-8-caffeic ester, as well as many TFs including MYB and bHLH. The UGFT enzyme of plants was the final gene in the flavonoid pathway. Their high accumulation level is essential for flavonoids and anthocyanin biosynthesis including gossypetin and quercetin biosynthesis (Zhao et al., 2012).

In the later research, it was reported that the brightness of the yellow color in *P. vulgaris* is largely determined by the carotenoid content (Cao and Liang, 2008). The major carotenoids detected in the yellow petals in *P. polyantha* and *P. helodoxa* were (9Z)-violaxanthin, (all-E)-violaxanthin, lutein, and antheraxanthin, which were present in esterified forms (Yamamizo et al., 2011). As expected, similar phenomena were found in HL petals in the present work. Correspondingly, the genes in the carotenoid biosynthetic pathway exhibited the highest expression levels in HL petals, including *PSY*, *PDS*, *Z-ISO*, *ZDS*, *LYCB*, *HYDB*, and *CYP97C* (Figure 7 and Supplementary Table S8). The carotenoid biosynthetic pathway diverges with the modification of all-trans-lycopene by lycopene ε-cyclase (*LYCE*) or *LYCB*. The expression level of *LYCB* was 60-fold higher than that of *LYCE*. Therefore, due to competition for the same substrate, β-cryptoxanthin and β-carotene were more abundantly accumulated than lutein.

Interestingly, the content of gossypetin derivatives was more abundant than that of the major carotenoids and had the highest positive correlation with b^∗^ indicating the balance between yellow and blue. In brief, the formation of the yellow flower color was derived from the combination of gossypetin and carotenoids, among which gossypetin might contribute more under the control of F3H, F3′H, and 3GT, and carotenoids potentially affected the color brightness under genes involved in the carotenoid biosynthetic pathway, and that this process was regulated by MYB and bHLH.

### White Color Formation] in *P. vulgaris*

None of the anthocyanidin types were identified as being involved in white color formation. The colorless quercetin- and kaempferol-related flavonols showed high accumulation levels in WL. In the coexpression network (Figure 8), four *CHS* (c36929, c14043.graph_c0, c14043.graph_c1, and c57559) and three *MYB* (c36093, c36140, and c41292) genes showed positive correlation with quercetin and kaempferol, and these *CHS* and *MYB* genes were coexpressed. Previous studies indicated that *CHS* played important roles in the biosynthesis of these achromatic quercetin- and kaempferol-related flavonols, and induced the changes in flower color via regulation by a pathway-specific MYB. Among the three MYBs, c36093 and c41292 upregulated in WL with more abundant expression levels of the four CHS. Thus, we speculated that *MYBs* (c36093 and c41292) functioned as hub genes that regulated or coexpressed with *CHS*, playing an important role in quercetin and kaempferol biosynthesis.

### The Transcriptional Regulation of MYBs Involved in Flower Coloration

In the coexpression network, several *MYBs* were considered as “hub genes” because of their high connectivity (Figure 8). In all species studied to date, flavonoid regulation is controlled by a transcriptional activation complex consisting of R2R3-MYB and bHLH TFs and a WD40 protein, which regulate the transcription of flavonoid biosynthetic genes (Hichri et al., 2011; Afrin et al., 2014). *c39130* (*MYB*) had 17 edges and was positively correlated with the accumulation of seven transcription factors, three carotenoid-genes, two carotenoid metabolites, two flavonoid genes, five flavonoid metabolites, and one anthocyanin gene. The top hub gene MYB-1 (*c44135*) in the BO network positively coexpressed with hirsutin, rosinin, petunidin, malvidin, which were the primary pigments in blue petals, and upregulated *CHS*, *F3H*, and *AOMT*. By contrast, *c45437* (*MYB*) had 14 edges and was negatively correlated with the accumulation of two transcription factors, five flavonoid metabolites, one flavonoid gene, four carotenoid metabolites, and two anthocyanin metabolites. The other *R2R3-MYB* unigene shared positive or negative correlations with *CHS*, *CHI*, *DFR*, *MATE*, and *bHLH* (Figure 8). In *Ginkgo biloba*, the transcription of *GbMYB2* is negatively correlated with the flavonoid content, whereas in *Arabidopsis*, overexpression of *GbMYB2* is correlated with the inhibition of flavonoid and anthocyanin biosynthesis compared with the untransformed plants (Xu et al., 2014). In *Arabidopsis*, CPC is a negative regulator of anthocyanin biosynthesis (Zhu et al., 2009). These results indicate that many MYBs act not only as positive regulators but also as negative regulators in the flavonoid biosynthesis pathway. Therefore, we inferred that R2R3-MYB TFs regulated flower coloration by regulating the target genes *CHS*, *CHI*, *DFR*, and *MATE* in tandem with *bHLH.* These results were consistent with those of other reports of the transcriptional regulation of anthocyanin biosynthesis (Pattanaik et al., 2010; Li et al., 2016; Zhang et al., 2017).

The previous study showed that an R2R3-MYB TF, RCP1, can simultaneously activate carotenoid biosynthetic genes and repress the expression of an anthocyanin-activating MYB TF in M. lewisii (Zhou et al., 2019). In the current study, the hub genes *c40864* (*MYB-2*), *c48106* (*MYB*), *c39130* (*MYB-3*), *c47013* (*MYB*), and *c50758* (*bHLH*) showed higher expression levels in HL and positively coexpressed with many carotenoid biosynthetic genes and carotenoid metabolites, and meanwhile positively coexpressed with quercetagetin and gossypetin, and their biosynthetic gene *F3H*. Following transient expression experiment of *c40864* (*MYB-2*) and *c39130* (*MYB-3*) in *Primula* protoplast confirmed our bioinformatics analysis result (Figure 9B). Thus, a potential direct link between the flavonoid and carotenoid pathways exists in the form of R2R3-MYB TF regulation. However, this speculation requires investigation in further research.

## Conclusion

In conclusion, although the petal epidermis structure was significantly different among petals of different colors and was partly contributed to final flower colors, different pigment combinations and their different accumulations were the primary causes of the different coloration in *P*. *vulgaris*. Hirsutin, rosinin, petunidin, malvidin, and the copigment herbacetin contributed to the blue coloration, while peonidin and delphinidin showed high accumulation levels in pink flowers, mainly via *CHS* (*c46201*), *CHI* (*c40107*), *FLS* (*c15244*), *DFR* (*c50255*), *ANS* (*c48970*), *3GT* (*c50034*), and *AOMT* (*c47583*). Yellow coloration was mainly due to gossypetin associated with carotenoids affecting the color brightness by *F3H* (*c43100*), *F3′H* (*c53714*), and *3GT* (*53907*) in addition to carotenoid biosynthetic pathway-related genes. Furthermore, we also identified a candidate direct link between anthocyanin biosynthetic pathway and carotenoid biosynthetic pathway through R2R3-MYB TFs. This work reveals the pigmentation mechanisms for *Primula*. This subject is probably more complex than what has been described here; thus, its elucidation could be an interesting and challenging subject for future research.

## Data Availability Statement

The datasets presented in this study can be found in online repositories. The names of the repository/repositories and accession number(s) can be found in the article/Supplementary Material.

## Author Contributions

LL performed the experiments and data analysis and wrote the manuscript. JY participated in some experiments and revised the manuscript. HL helped prepare the plant material and performed some experiments for this manuscript. LL and QS designed the research. All authors read and approved the final manuscript.

## Conflict of Interest

The authors declare that the research was conducted in the absence of any commercial or financial relationships that could be construed as a potential conflict of interest.

## Footnotes

1 https://www.r-project.org/

## Supplementary Material

Supplementary Figure 1 Protoplasts from the petals of the four cultivars. **(A)** Protoplasts from upper epidermis. **(B)** Protoplasts from lower epidermis. Bars, 30 μm. WL, HL, BS, and BO represent “White Lover,” “Huang Li,” “Beautiful Scenery,” and “Blue Onstar,” respectively.

Supplementary Figure 2 Chromatograms of anthocyanin extracts from differentially colored petals and the standards. HPLC-DAD-HRMS analysis under 520 nm of anthocyanin extracts from differentially colored petals. The substances (7–15) without chromatograms of the standard were referenced to Harborne (1968) and Venter et al. (2013). Nos. 1–15 represent the identified fifteen components of anthocyanins. WL, HL, BS, and BO represent “White Lover,” “Huang Li,” “Beautiful Scenery,” and “Blue Onstar,” respectively.

Supplementary Figure 3 Chromatograms of flavonols extracts from differentially colored petals and the standards. **(A)** HPLC-DAD-HRMS analysis under 360 nm of flavonols extracts from differentially colored petals. **(B)** Chromatograms of the standards were shown on **(B)**. The substances (4–20) without chromatograms of the standard were referenced to Harborne (1965, 1968). Nos. 1–20 represent the identified fifteen components of flavonols. WL, HL, BS, and BO represent “White Lover,” “Huang Li,” “Beautiful Scenery,” and “Blue Onstar,” respectively.

Supplementary Figure 4 Chromatograms of carotenoid extracts from differentially colored petals and the standards. **(A)** HPLC-DAD-HRMS analysis under 450 nm of carotenoid extracts from differentially colored petals. **(B)** Chromatograms of the standards are shown in **(B)**. The substances (1, 13, and 3–11) without chromatograms of the standard were referenced to Harborne (1965, 1968) and Venter et al. (2013). WL, HL, BS, and BO represent “White Lover,” “Huang Li,” “Beautiful Scenery,” and “Blue Onstar,” respectively.

Supplementary Figure 5 Differentially expressed genes related to flower coloration in *Primula*. **(A)** Overlapping sets of up-regulated unigenes in yellow, pink, and blue cultivars. **(B)** Overlapping sets of repressed unigenes in yellow, pink, and blue cultivars. The white-flowered cultivar “White Lover” was used as a control. HL, BS, and BO represent “Huang Li,” “Beautiful Scenery,” and “Blue Onstar,” respectively.

Supplementary Figure 6 The coloring of the q-values indicates the significance of the richness factor, ranging from 0 to 1, and a lower *q*-value indicates greater intensity. The richness factor is the ratio of DEG numbers annotated in a given pathway term to all gene numbers annotated in that pathway term. A higher richness factor indicates greater intensiveness. The top 20 pathway terms enriched according to the KEGG database are listed in this figure. The circle size represents the quantity of DEGs. **(A)** “Huang Li” vs. “White Lover”; **(B)** “Beautiful Scenery” vs. “White Lover”; **(C)** “Blue Onstar” vs. “White Lover”.

Supplementary Figure 7 Verification of DGEs by qRT-PCR. The relative amount of mRNA (*y*-axis) is a ratio normalized by TIP41 (tonoplast intrinsic protein 41). WL, HL, BS, and BO represent “White Lover,” “Huang Li,” “Beautiful Scenery,” and “Blue Onstar,” respectively.

Supplementary Figure 8 Correlation of gene expression results obtained from qRT-PCR analysis and RNA-seq for color-related genes in four cultivars of *Primula vulgaris* with different flower colors.

Supplementary Figure 9 Phylogenetic tree of MYB transcription factors showing the relationship between *Arabidopsis* MYBs and *Primula vulgaris* MYB sequences in this study.

Supplementary Figure 10 The relative expression level of GFP label among different protoplast transformation experiment.

Supplementary Table 1 The contents of anthocyanins in petals of the four different flower colored cultivars of *Primula vulgaris* at fully open stage.

Supplementary Table 2 The contents of glycosides of flavonol in petals of the four different flower colored cultivars of *Primula vulgaris* at fully open stage.

Supplementary Table 3 The contents of carotenoids in petals of the four different flower colored cultivars of *Primula vulgaris* at fully open stage.

Supplementary Table 4 The Pearson correlation between the content of pigments and parameters of flower color in four cultivars of *Primula vulgaris.*

Supplementary Table 5 Statistics of functional annotation.

Supplementary Table 6 Putative unigenes related to flower pigmentation.

Supplementary Table 7 Expression profiles of differentially expressed genes involved in flower pigmentation in *Primula vulgaris* cultivars.

Supplementary Table 8 Putative unigenes related to transcriptional regulation and transferase activity in *Primula vulgaris.*

Supplementary Table 9 Three transcription factor families differentially expressed in different-colored petals.

Supplementary Table 10 List of primers used for qRT-PCR analysis.

Supplementary Table 11 List of primers used for gene cloning.
