# Supplementary material for: Characterization of Metabolites and Transcripts Involved in Flower Pigmentation in Primula vulgaris
Source: Front Plant Sci. 2020 Nov 20;11:572517. doi: 10.3389/fpls.2020.572517 (PMC7714730; doi:10.3389/fpls.2020.572517)
Supplement: Supplementary Figure 1 — Protoplasts from the petals of the four cultivars. (A) Protoplasts from upper epidermis. (B) Protoplasts from lower epidermis. Bars, 30 μm. WL, HL, BS, and BO represent “White Lover,” “Huang Li,” “Beautiful Scenery,” and “Blue Onstar,” respectively. [file Data_Sheet_1.PDF]

Characterization of metabolites and transcripts involved in flower pigmentation in *Primula vulgaris*

Long Li[1, †], Jing Ye[1, †], Houhua Li[2], Qianqian Shi[2*]

[1] College of Forestry, Northwest A&F University, Yangling 712100, Shaanxi, China; [2] College of Landscape Architecture and Art, Northwest A&F University, Yangling 712100, Shaanxi, China
Lilong1949@126.com (L.L.); yejing@nwsuaf.edu.cn(J.Y.); lihouhua73@163.com (H.L.).

* Corresponding author: Email: shiqianqian2005@163.com (Qianqian Shi). Tel.: +86-29-8708-2878 (Qianqian Shi)
† Equal contribution.

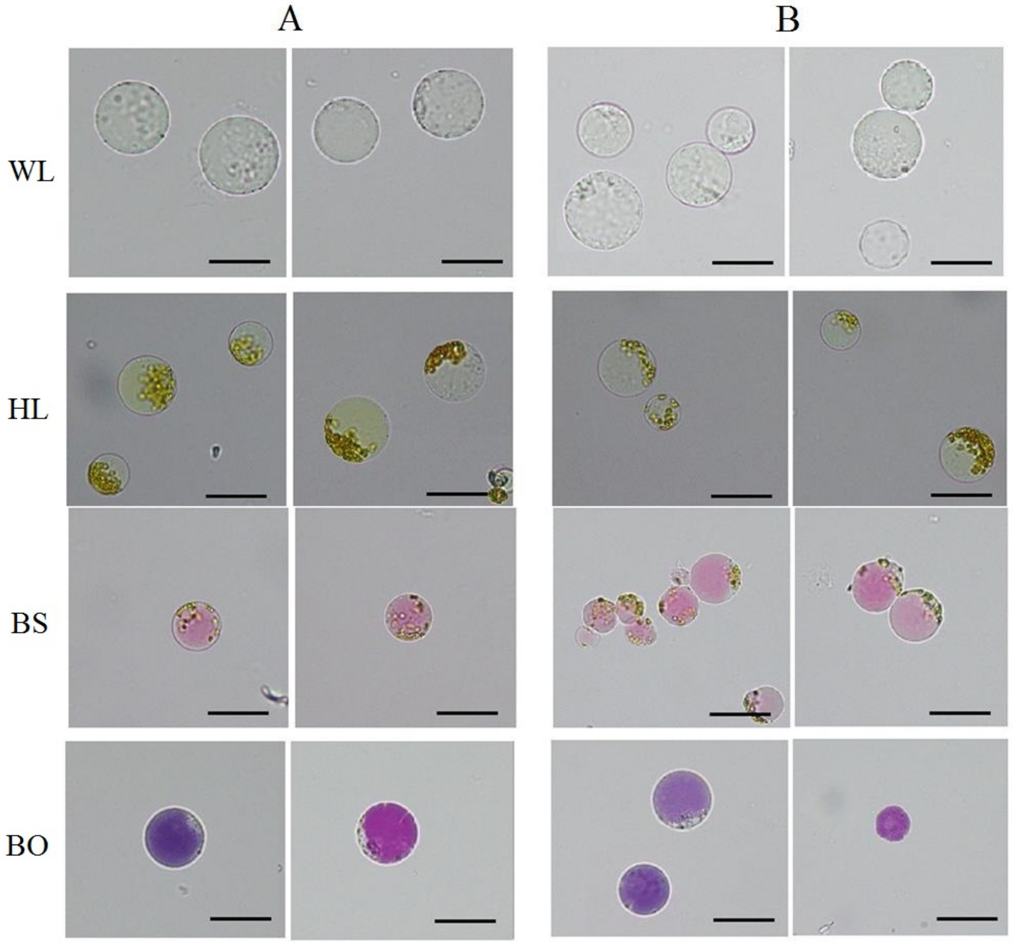

Figure S1. Protoplasts from the petals of the four cultivars. (A) Protoplasts from upper epidermis. (B) Protoplasts from lower epidermis. Bars, 30 μm. WL, HL, BS and BO represent ‘White Lover’, ‘Huang Li’, ‘Beautiful Scenery’ and ‘Blue Onstar’, respectively.

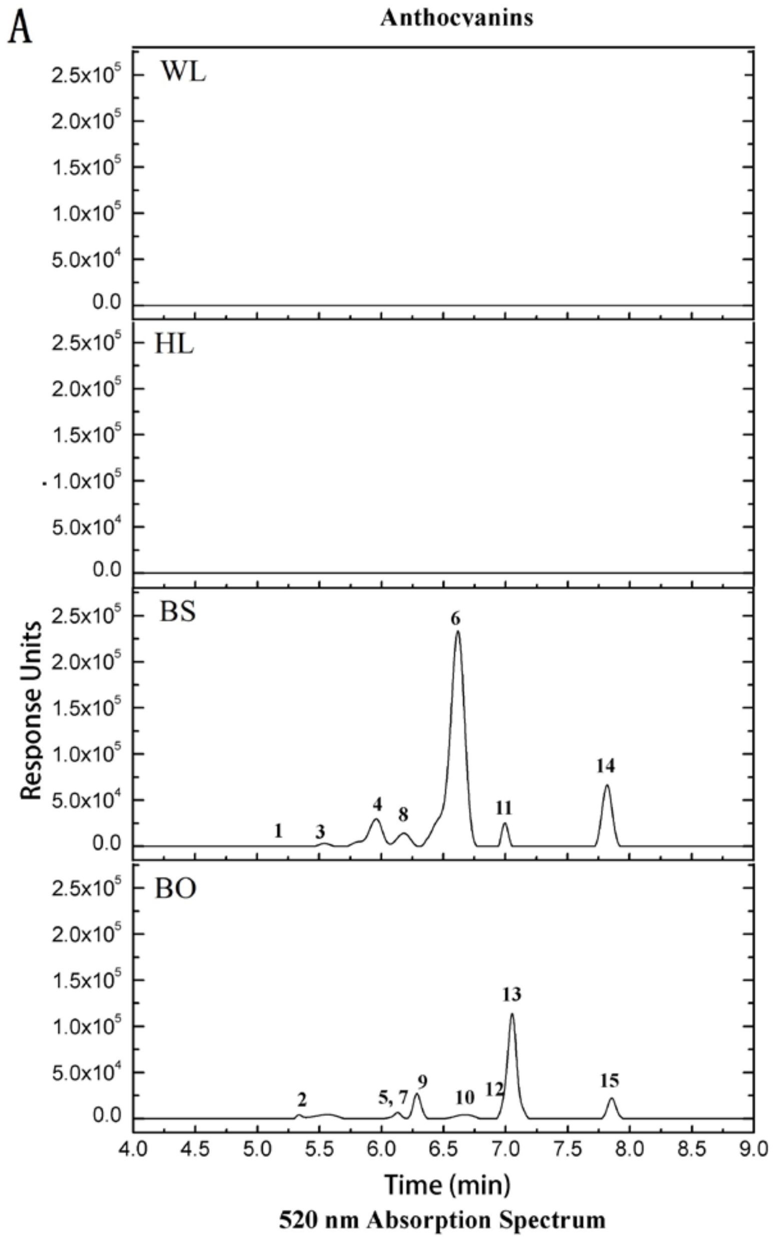

Figure S2. Chromatograms of anthocyanin extracts from differentially colored petals and the standards. (A) HPLC-DAD-HRMS analysis under 520 nm of anthocyanin extracts from differentially colored petals. The substances (7-15) without chromatograms of the standard were referenced to Harborne JB (1968) and Venter et al. (2013). Nos. 1-15 represent the identified fifteen components of anthocyanins. WL, HL, BS and BO represent ‘White Lover’, ‘Huang Li’, ‘Beautiful Scenery’ and ‘Blue Onstar’, respectively.

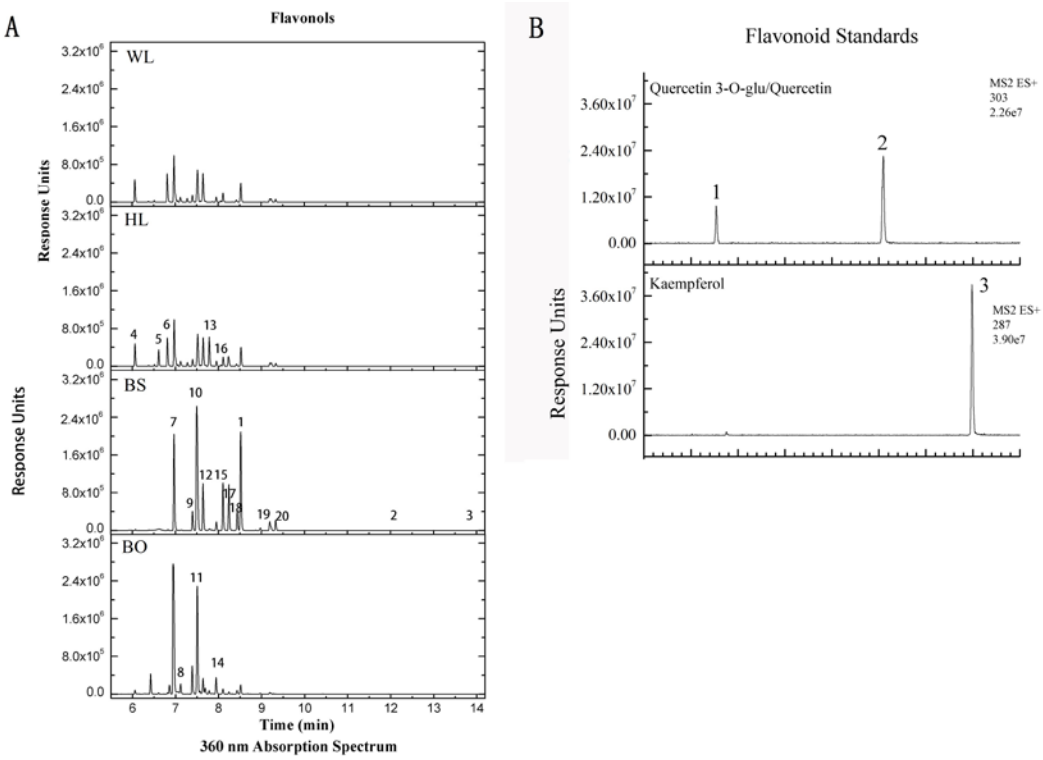

Figure S3. Chromatograms of flavonoids extracts from differentially colored petals and the standards. (A) HPLC-DAD-HRMS analysis under 360 nm of flavonoids extracts from differentially colored petals. (B) Chromatograms of the standards were shown on panel B. The substances (4-20) without chromatograms of the standard were referenced to Harborne JB (1968) and Harborne JB (1965). Nos. 1-20 represent the identified fifteen components of flavonols and flavones. WL, HL, BS and BO represent 'White Lover', 'Huang Li', 'Beautiful Scenery' and 'Blue Onstar', respectively.

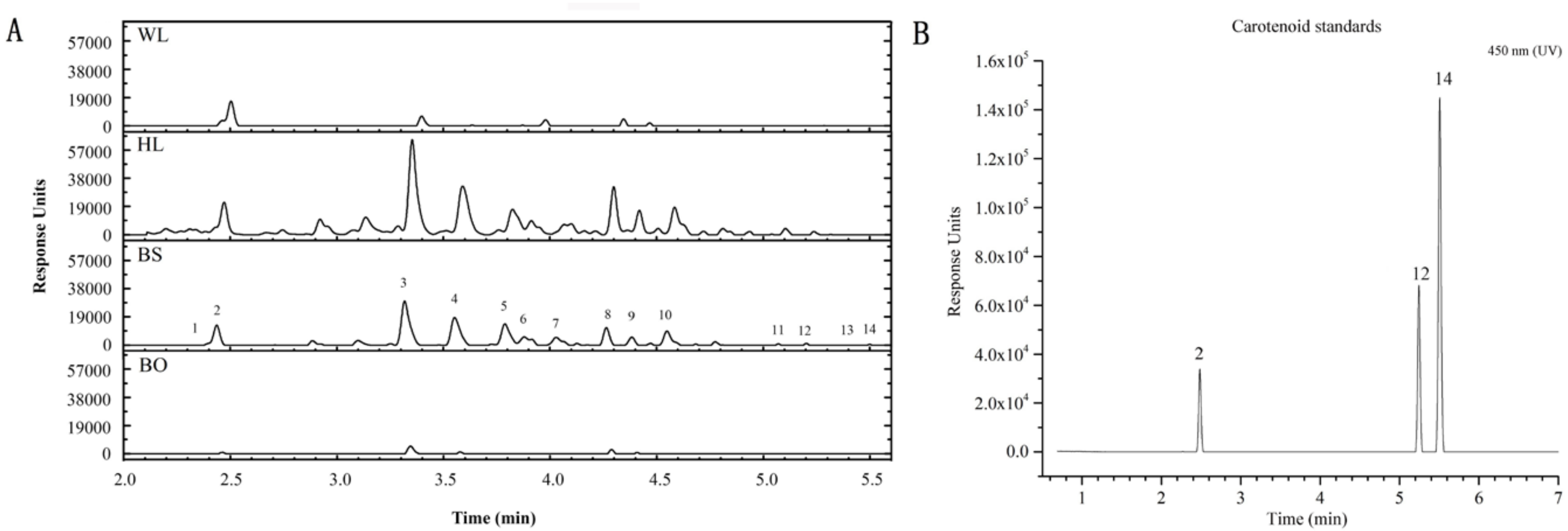

Figure S4. Chromatograms of carotenoid extracts from differentially colored petals and the standards. (A) HPLC-DAD-HRMS analysis under 450 nm of carotenoid extracts from differentially colored petals. (B) Chromatograms of the standards were shown panel B. The substances (1, 13 and 3-11) without chromatograms of the standard were referenced to Harborne JB (1968), Harborne JB (1965) and Venter et al. (2013). WL, HL, BS and BO represent ‘White Lover’, ‘Huang Li’, ‘Beautiful Scenery’ and ‘Blue Onstar’, respectively.

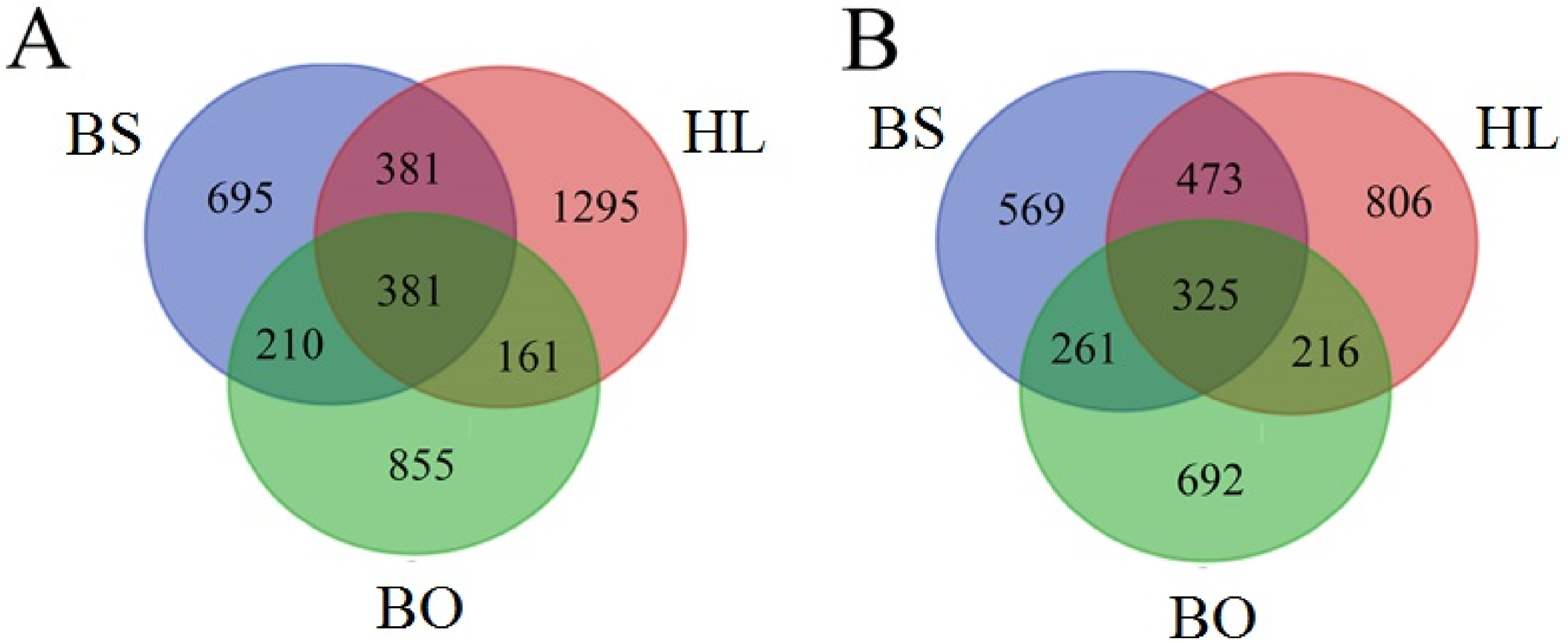

Figure S5. Differentially expressed genes related to flower coloration in *Primula*. (A) Overlapping sets of up-regulated unigenes in yellow, pink and blue cultivars. (B) Overlapping sets of repressed unigenes in yellow, pink and blue cultivars. The white-flowered cultivar ‘White Lover’ was used as a control. HL, BS and BO represent ‘Huang Li’, ‘Beautiful Scenery’ and ‘Blue Onstar’, respectively.

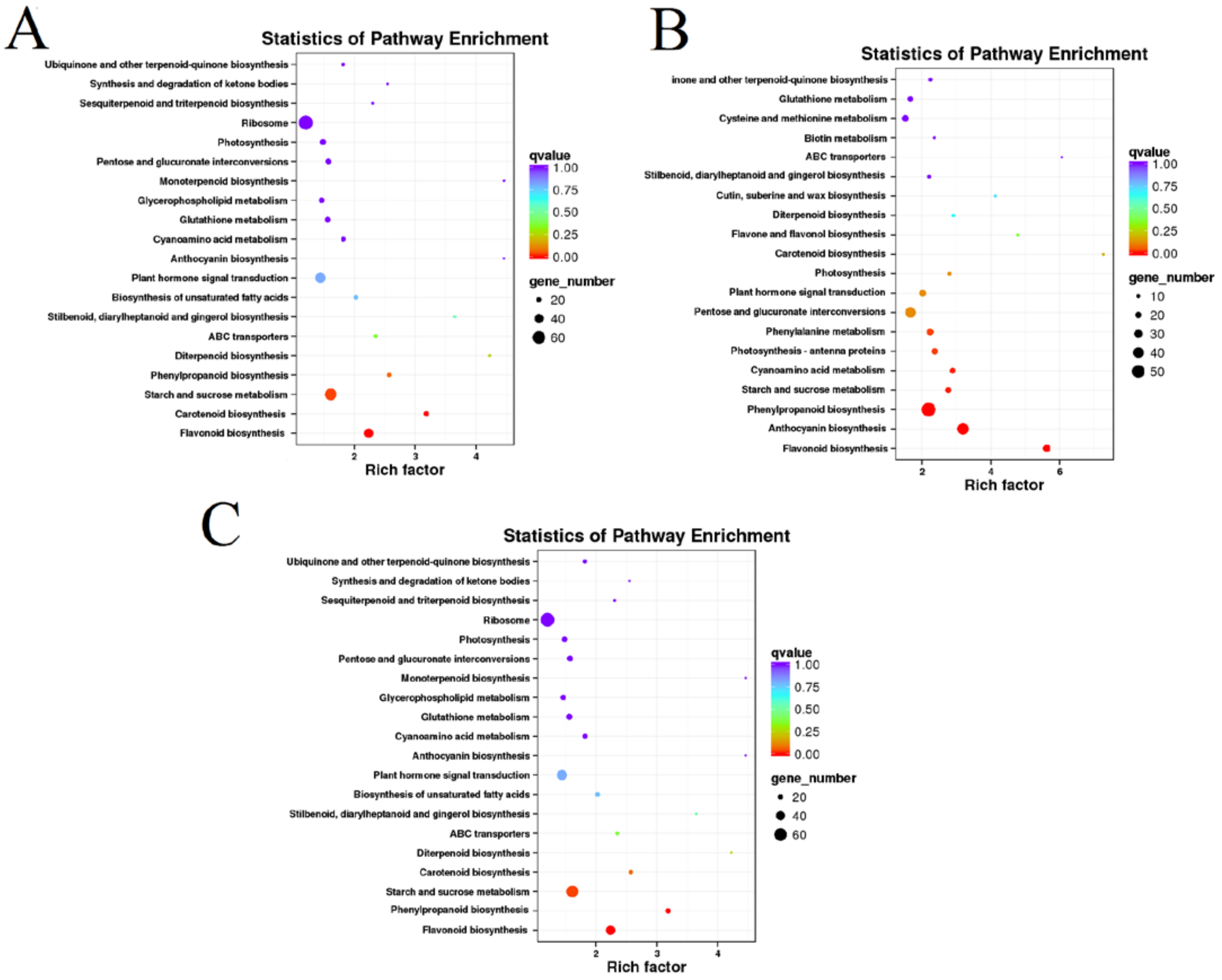

Figure S6. The coloring of the q-values indicates the significance of the richness factor, ranging from 0 to 1, and a lower q-value indicates greater intensity. The richness factor is the ratio of DEG numbers annotated in a given pathway term to all gene numbers annotated in that pathway term. A higher richness factor indicates greater intensiveness. The top 20 pathway terms enriched according to the KEGG database are listed in this figure. The circle size represents the quantity of DEGs. (A) ‘Huang Li’ vs ‘White Lover’; (B) ‘Beautiful Scenery’ vs ‘White Lover’; (C) ‘Blue Onstar’ vs ‘White Lover’.

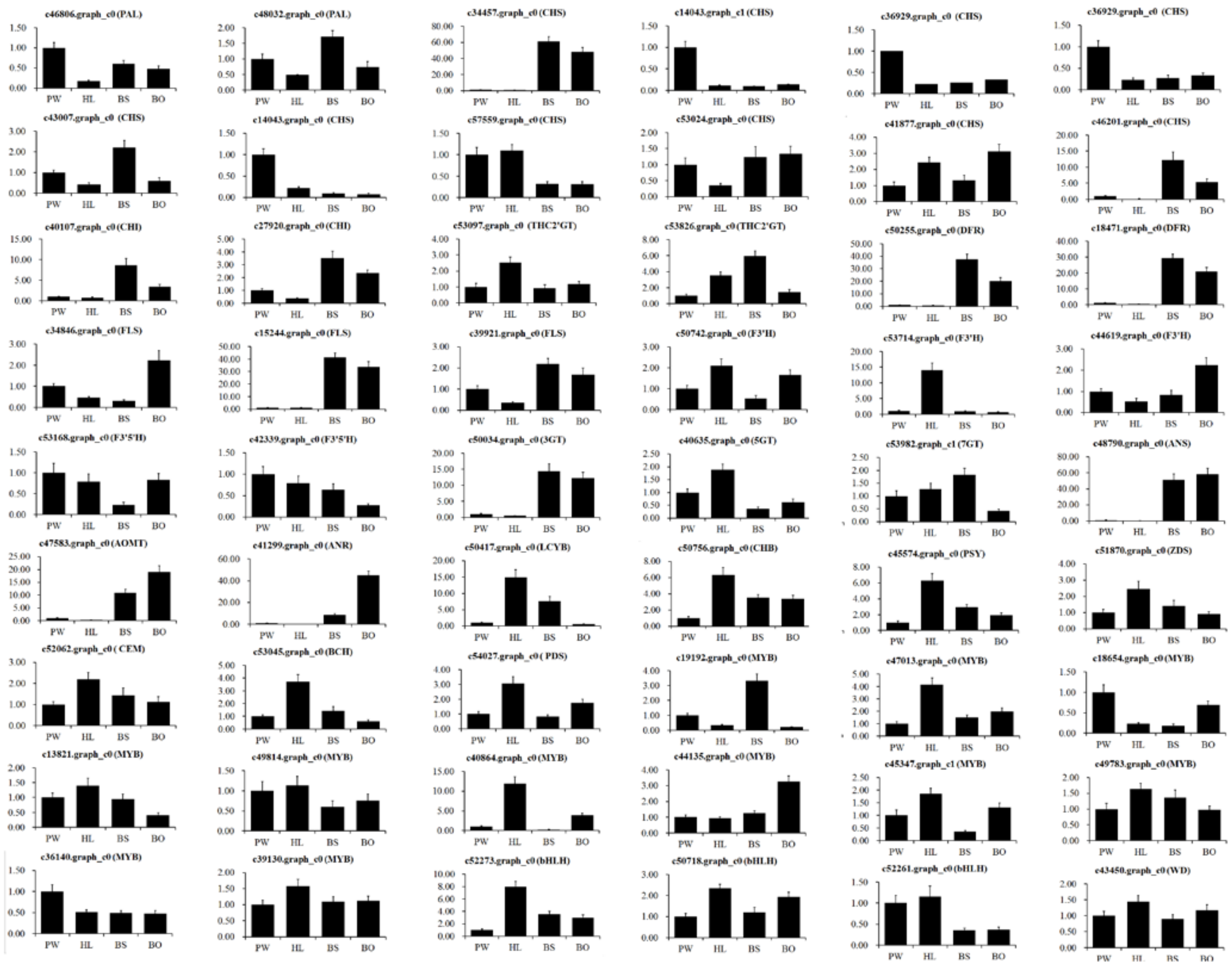

Figure S7. Verification of DGEs by qRT-PCR. The relative amount of mRNA (y-axis) is a ratio normalized by *TIP41* (tonoplast intrinsic protein 41). WL, HL, BS and BO represent ‘White Lover’, ‘Huang Li’, ‘Beautiful Scenery’ and ‘Blue Onstar’, respectively.

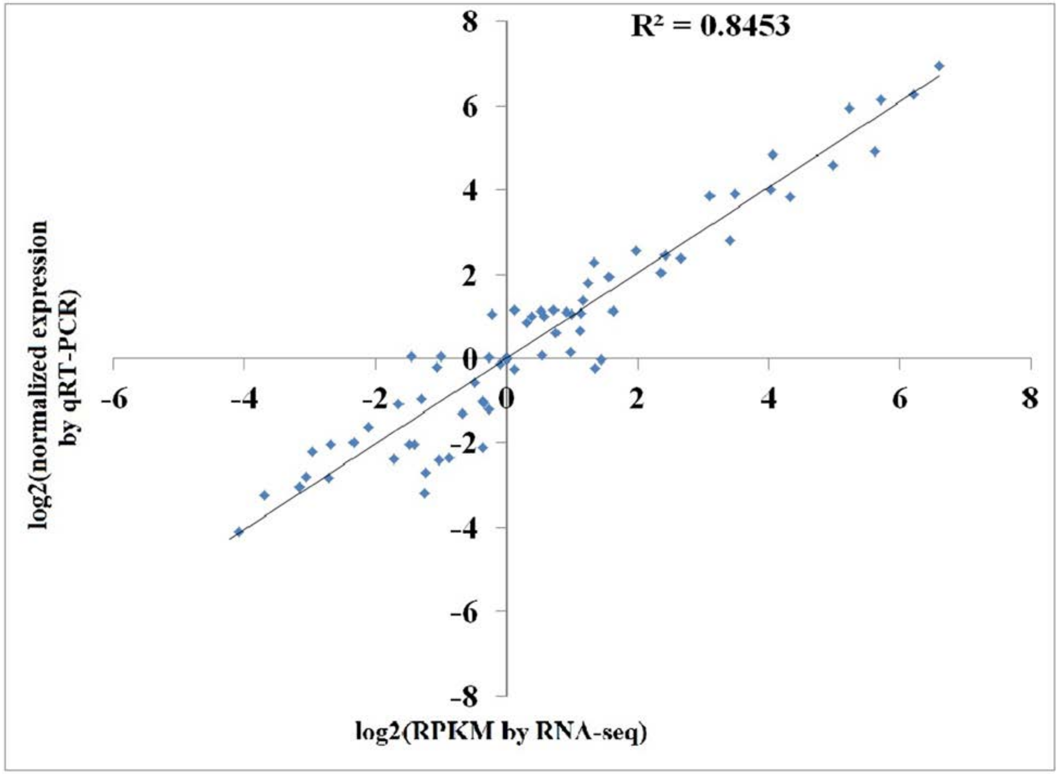

Figure S8. Correlation of gene expression results obtained from qRT-PCR analysis and RNA-seq for color-related genes in four cultivars of Primula vulgaris with different flower colors.

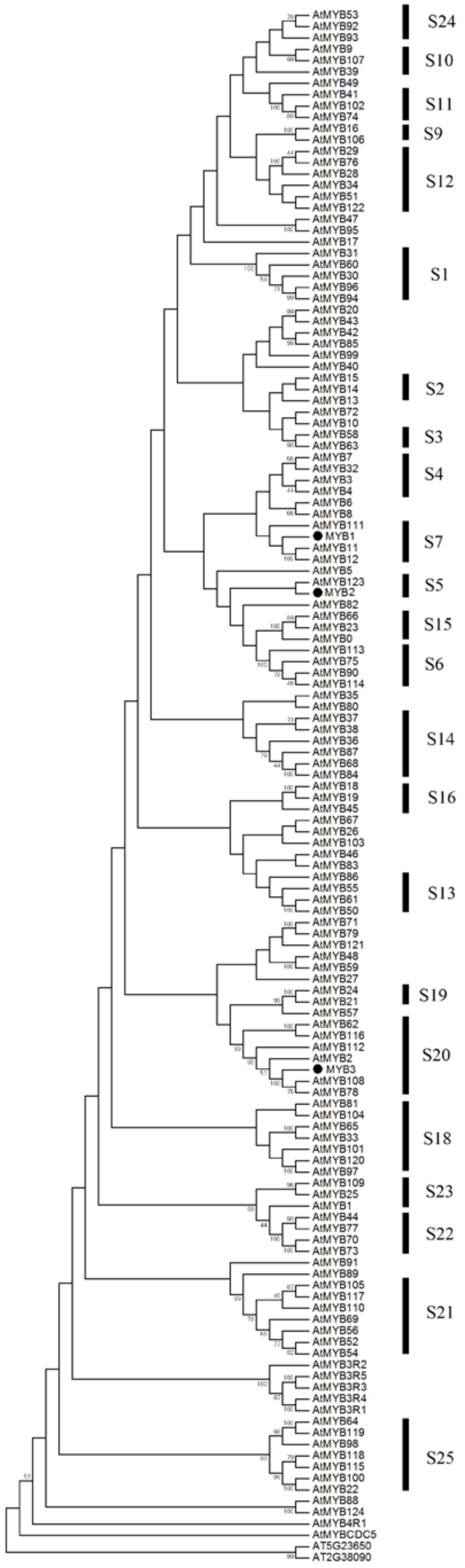

Figure S9. Phylogenetic tree of MYB transcription factors showing the relationship between Arabidopsis MYBs and Primula vulgaris MYB sequences in this

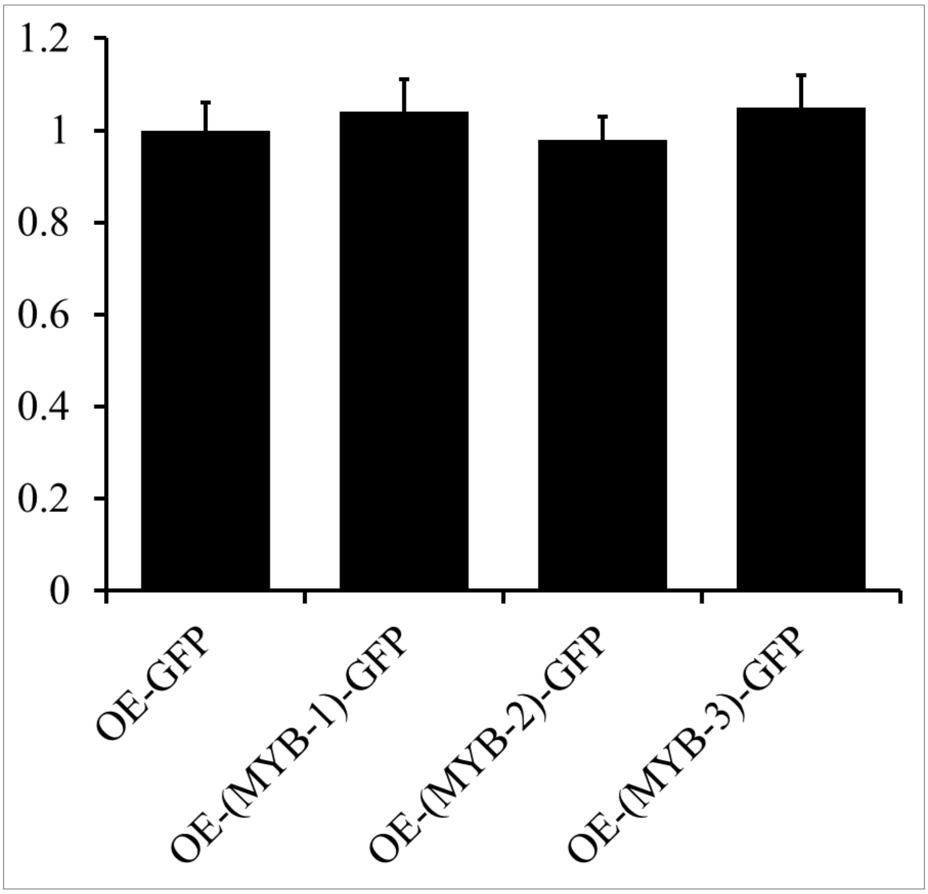

Figure S10. The relative expression level of GFP label among different protoplast transformation experiment.

Table S1. The contents of anthocyanins in petals of the four different flower colored cultivars of *Primula vulgaris* at fully open stage.

**Peak no.**	**Compound**	**Retention time (min)**	**λmax （nm）**	**Molecular ion(m/z)**	**Fragment ions (m/z)**	**Content**			
						**WL[a](mg/g)**	**HL[a] (mg/g)**	**BS[a] (mg/g)**	**BO[a] (mg/g)**
1	Pelargonidin 3,5-di-*O*-glucoside	5.34	280, 520	595	433, 271	0.0±0.0	0.0688±0.0025	0.0153±0.0012	0.0264±0.003
2	Delphinidin 3-*O*-glucoside	5.46	280, 521	465	303	0.0±0.0	0.0±0.0	0.0±0.0	0.318±0.025
3	Peonidin 3,5-di-*O*-glucoside	5.54	280, 522	625	301	0.0±0.0	0.0±0.0	7.617±0.048	0.326±0.017
4	Cyanidin 3-*O*-glucoside	5.96	280, 524	449	287	0.0±0.0	0.0035±0.0009	7.976±0.054	0.0884±0.007
5	Petunidin 3-*O*-glucoside	6.16	280, 525	479	317	0.0±0.0	0.0±0.0	0.0±0.0	0.993±0.048
6	Peonidin 3-*O*-glucoside	6.62	280, 526	463	301	0.0±0.0	0.0276±0.0019	24.923±1.839	0.075±0.013
7	Peonidin 3- *O*-sophoroside	6.13	280, 524	625	301	0.0±0.0	0.0±0.0	0.0±0.0	1.653±0.130
8	Peonidin 3- *O*-gentiobiosiden	6.18	280, 522	625	301	0.0±0.0	0.0±0.0	13.641±0.798	0.0±0.0
9	Petunidin 7-methoxy-3,5-di-*O*-glucoside	6.28	280, 523	655	479/349/317	0.0±0.0	0.0±0.0	0.0±0.0	5.459±0.4801
10	Malvidin 3-*O*-glucoside	6.67	280, 524	493	331	0.0±0.0	0.0±0.0	0.0±0.0	2.814±0.378
11	Delphininidin 3,5-di-*O*-glucoside-3′-caffeic ester	6.99	280, 522	789	627/465/303	0.0±0.0	0.0±0.0	6.562±0.456	0.0±0.0
12	Rosinin	7.01	280, 523	639	477/ 315	0.0±0.0	0.0±0.0	0.0±0.0	1.736±0.105
13	Hirsutin	7.05	280, 522	669	507/ 345	0.0±0.0	0.0±0.0	0.0±0.0	27.961±1.482
14	Peonidin 7-methoxy-3-*O*-glucoside	7.82	280, 523	477	315	0.0±0.0	0.0±0.0	12.048±1.760	0.0±0.0
15	Hirsutin 4’-malonate	7.85	280, 524	755	507/363/345	0.0±0.0	0.0±0.0	0.0±0.0	8.115±0.745

[a] WL, HL, BS and BO represent ‘White Lover’, ‘Huang Li’, ‘Beautiful Scenery’ and ‘Blue Onstar’.

Table S2. The contents of glycosides of flavonoids in petals of the four different flower colored cultivars of *Primula vulgaris* at fully open stage.

**Peak no.**	**Compound**	**Retention time (min)**	**λmax （nm）**	**Molecular ion(m/z)**	**Fragment ions (m/z)**	**Content**			
						**WL[a] (mg/g)**	**HL[a] (mg/g)**	**BS[a] (mg/g)**	**BO[a] (mg/g)**
1	Quercetin 3-*O*-glucoside	8.52	255, 353	465	303	0.3189±0.0208	0.6683±0.0245	1.8282±0.1351	0.2639±0.0203
2	Quercetin	12.09	254, 370	303	—	0.7785±0.0219	0.1337±0.0124	0.1276±0.0090	0.1358±0.0129
3	kaempferol	13.99	264, 365	287	—	0.5792±0.0468	0.2038±0.0189	0.0323±0.0018	0.0468±0.0027
4	Gossypetin 3,5-di-*O*-glucoside-8-caffeic ester	6.06	255, 353	805	643/ 481/ 319	0.0132±0.0012	0.6576±0.0309	0.0381±0.0029	0.1775±0.0129
5	Quercetin 3- gentiobiosiden -7-*O*-glucoside	6.61	255, 353	789	627/465/ 303	0.0±0.0	2.2002±0.1375	0.0221±0.0012	0.2727±0.0201
6	Gossypetin 7-methoxy-3,5-di-*O*-glucoside-8-caffeic ester	6.84	279, 343	819	657/ 495/333	0.0118±0.0	3.3304±0.2115	0.2528±0.0234	0.2795±0.0251
7	Quercetin 3- gentiobiosiden -5-*O*-glucoside	6.97	255, 353	789	627/465/303	0.4985±0.0380	2.6899±0.1975	3.0527±0.0139	4.2256±0.0165
8	Gossypetin 7-methoxy-3,5-di-*O*-glucoside-8-caffeic ester	7.12	274, 361	819	657/ 495/333	0.1894±0.0139	0.7270±0.0263	0.6753±0.0326	0.3513±0.0281
9	Quercetin 3- gentiobiosiden-acetate-7-*O*-glucoside	7.4	255, 353	831	465/ 367/ 303	2.988±0.2008	1.1226±0.0858	2.051±0.1394	2.4834±0.2086

10	Quercetin 3,5-di-*O*-glucoside	7.51	255, 353	627	465/ 303	0.9250±0.0831	0.8436±0.0723	3.6785±0.2509	0.456±0.0382
11	Kaempferol 3-*O*-gentiotrioside	7.55	255, 353	773	611/ 449/287	4.2069±0.2078	1.9292±0.1085	1.4008±0.0776	3.8029±0.1918
12	Quercetin 7-methoxy-3-*O*-gentiotrioside	7.64	255, 353	803	641/ 479/ 317	3.298±0.1609	2.4289±0.1642	2.4416±0.1835	1.1374±0.0689
13	Gossypetin 7,3′-dimethoxy-3,5-di-*O*-glucoside-8-caffeic ester	7.79	255, 353	833	671/ 509/ 347	1.6852±0.1246	2.2861±0.1782	0.01216±0.0083	0.2479±0.0216
14	Herbacetin 3- rutinoside-5-*O*-glucoside	7.95	255, 353	815	653/ 507/ 345	0.0±0.0	0.9812±0.0376	1.4193±0.1073	1.9806±0.1124
15	Kaempferol 3,5- di-*O*-glucoside	8.12	255, 353	611	449/ 287	0.3408±0.0289	0.2312±0.0125	1.7352±0.1254	0.2002±0.0118
16	Quercetin 7-methoxy-3-*O*-glucoside-5-sophoroside-acetate	8.12	255, 353	845	641/ 479/317	2.8501±0.1895	1.6063±0.1332	1.1439±0.0702	0.4043±0.0253
17	Isorhamnetin 3-*O*-glucoside-caffeic ester	8.24	255, 353	641	479/ 317	3.9582±0.2019	0.3162±0.0185	2.2949±0.1079	0.0666±0.0048
18	Gossypetin 7-methoxy-3-*O*-glucoside	8.42	255, 353	495	333	0.0±0.0	0.1535±0.0114	0.0219±0.0013	0.021±0.0006
19	Kaempferol 3-*O*-glucoside	9.2	255, 353	449	287	0.0779±0.0139	0.1393±0.0075	0.2146±0.0185	0.081±0.0068
20	Quercetin 7-methoxy -3-*O*-glucoside	9.34	255, 353	479	317	0.0875±0.0054	0.1548±0.012	0.2699±0.0161	0.0355±0.0193

[a] WL, HL, BS and BO represent ‘White Lover’, ‘Huang Li’, ‘Beautiful Scenery’ and ‘Blue Onstar’.

Table S3. The contents of carotenoids in petals of the four different flower colored cultivars of *Primula vulgaris* at fully open stage.

**Peak no.**	**Compound**	**Retention time (min)**	**Λmax (mm)**	**Molecular ion (m/z)**	**Content**			
					**WL[a](mg/g)**	**HL[a] (mg/g)**	**BS[a] (mg/g)**	**BO[a] (mg/g)**
1	a-Carotene	2.34	441, 471	536[M]$^+$	0.0±0.0	0.061±0.004	0.0±0.0	0.0±0.0
2	β-Carotene	2.51	435, 461, 270	536[M]$^+$	0.051±0.001	1.046±0.098	0.715±0.026	0.121±0.008
3	Acetyl-Lutein	3.4	402, 423, 450	611[M+H]$^+$	0.083±0.002	0.642±0.012	0.203±0.002	0.0±0.0
4	Antheraxanthin dimethyl ester	3.64	425, 452	613[M+H]$^+$	0.031±0.012	2.569±0.284	1.314±0.086	0.146±0.034
5	Acetyl-zeaxanthin	3.87	424, 451	611[M+H]$^+$	0.014±0.007	1.808±0.145	0.979±0.090	0.0±0.0
6	Violaxanthin dimethyl ester	3.98	429, 453	629[M+H]$^+$	0.061±0.006	0.611±0.074	0.536±0.031	0.0±0.0
7	Lutein methyl ester	4.35	401, 425, 451	583[M+H]$^+$	0.016±0.001	1.264±0.346	0.538±0.083	0.223±0.03
8	Zeaxanthin methyl ester	4.47	401, 425, 451	583[M+H]$^+$	0.057±0.004	0.571±0.025	0.245±0.025	0.125±0.01
9	Cryptoxanthin methyl ester	4.64	428, 455	567[M+H]$^+$	0.031±0.003	1.030±0.104	0.574±0.009	0.0±0.0
10	lutein-5,6-epoxide dimethyl ester	4.99	425, 451	613[M+H]$^+$	0.0±0.0	0.167±0.013	0.0±0.0	0.0±0.0
11	(all E)violaxanthin/ (9Z) violaxanthin	5.16	429, 456	601[M+H]$^+$	0.023±0.002	0.312±0.004	0.135±0.012	0.0±0.0
12	lutein	5.29	432, 459 , 263	568[M]$^+$	0.061±0.006	0.257±0.013	0.225±0.031	0.0±0.0
13	Antheraxanthin	5.33	432, 461	584[M]$^+$	0.0±0.0	0.051±0.006	0.0±0.0	0.0±0.0
14	Zeaxanthin	5.59	438, 463 , 269	568[M]$^+$	0.0±0.0	0.101±0.010	0.193±0.031	0.0±0.0

[a] WL, HL, BS and BO represent ‘White Lover’, ‘Huang Li’, ‘Beautiful Scenery’ and ‘Blue Onstar’.

Table S4. The Pearson correlation between the content of pigments and parameters of flower color in four cultivars of *Primula vulgaris*.

	**Components**	**The correlation coefficient**		
		L*	***a****	***b****
Flavonoid	Quercetin	0.1508	0.6287	0.2518
	kaempferol	0.3902	-0.4943	-0.5256
	Gossypetin	0.2965	-0.2914	0.9219
	Herbacetin	-0.9951	0.6196	-0.5139
	Isorhamnetin	0.7128	-0.1951	-0.0227
Anthocyanin	Pelargonidin	-0.1974	0.0114	0.6531
	Delphinidin	-0.6828	-0.0385	-0.7057
	Peonidin	-0.3648	0.9159	-0.2589
	Cyanidin	-0.3455	0.9099	-0.2398
	Petunidin	-0.6828	-0.0385	-0.7057
	Malvidin	-0.6828	-0.0385	-0.7057
	Rosinin	-0.6828	-0.0385	-0.7057
	Hirsutin	-0.6828	-0.0385	-0.7057
Carotenoid	lutein	0.0244	0.1706	0.8346
	a-Carotene	0.1676	-0.1771	0.8825
	b-Carotene	-0.1092	0.4418	0.7045
	Antheraxanthin	-0.0317	0.2896	0.7882
	zeaxanthin	-0.0374	0.3433	0.7700
	Violaxanthin	0.0003	0.4308	0.7389
	Cryptoxanthin	0.0059	0.3226	0.7922

Table S5. Statistics of functional annotation.

Annotated Database	Annotated_Number	300<=length<1000	length>=1000
nr Annotation	45173(44.68%)	19155(18.94%)	16389(16.21%)
Pfam Annotation	31044(30.70%)	11982(11.85%)	14279(14.12%)
Swissprot Annotation	29231(28.91%)	11933(11.80%)	12037(11.90%)
COG Annotation	14514 (14.35%)	5316(5.26%)	6764(6.69%)
GO Annotation	27020(26.72%)	11609(11.48%)	9978(9.87%)
KEGG Annotation	18728(18.52%)	8107(8.02%)	6691(6.62%)
KOG Annotation	27202(26.90%)	11190(11.07%)	10359(10.25%)
All Annotated	46829(46.31%)	19682(19.47%)	16476(16.29%)
All Unigenes	101112 (100.00%）		

Table S6. Putative unigenes related to flower pigmentation.

**Pathway**	**Gene**	**Enzyme**	**Unigene number**
Flavonoid biosynthesis	CHS	Chalcone synthase	20
	CHI	Chalcone isomerase	3
	F3H	Flavanone 3-hydroxylase	4
	F3'H	Flavanone 3'-hydroxylase	5
	F3'5'H	Flavonoid 3',5'-hydroxylase	5
	FLS	Flavonol synthase	6
	DFR	Dihydroflavonol-4-reductase	5
Anthocyanin biosynthesis	ANS	Anthocyanidin synthase	1
	AOMT	Anthocyanin *O*-methyltransferase	3
	3GT	Anthocyanin 3-*O*-glycosyltransferase	13
	5GT	Anthocyanin 5-*O*-glycosyltransferase	1
	7GT	Anthocyanin 7-*O*-glycosyltransferase	1
Carotenoid biosynthesis	PSY	Phytoene synthase	4
	PDS	Phytoene desaturase	2
	Z-ISO	ε-carotene isomerase	1
	ZDS	ε-carotene desaturase	1
	CRTISO	carotenoid isomerase	3
	LYCB	lycopene β-cyclase	2
	LYCE	lycopene ε-cyclase	1
	CYP97C	carotene ε-ring hydroxylase	1
	HYDB	β-carotene hydroxylase	1
	ZEP	Zeaxanthin epoxidase	6
	VDE	Violaxanthin de-epoxidase	1

Table S7. Expression profiles of differentially expressed genes involved in flower pigmentation in *Primula vulgaris* cultivars.

Gene name	Unigene ID	FRKM			
		PW[a]	PY[a]	PP[a]	PB[a]
CHS	c14043.graph_c1	10.84	0.17	1.02	0.87
CHS	c36929.graph_c0	9.87	1.29	0.68	2.31
CHS	c43007.graph_c0	0.60	0.13	1.33	0.36
CHS	c14043.graph_c0	10.84	0.17	1.02	0.87
CHS	c57559.graph_c0	1.40	0.84	0.00	0.02
CHS	c53024.graph_c0	179.51	64.31	130.91	186.19
CHS	c41877.graph_c0	24.96	47.99	27.97	65.42
CHS	c46201.graph_c0	23.78	3.04	292.31	127.41
CHI	c40107.graph_c0	32.75	18.49	262.02	79.58
CHI	c27920.graph_c0	129.27	20.11	169.02	188.39
CHI	c53926.graph_c0	30.69	5.89	1922.47	898.96
CHI	c43168.graph_c0	6.83	0.91	930.75	136.97
CHI	c36576.graph_c0	0.937	5.92	1.08	0.35
CHI	c26605.graph_c0	0.42	2.75	0.30	0.20
F3H	c48030.graph_c0	66.10	124.80	65.78	73.45
F3H	c43100.graph_c0	18.56	359.66	7.32	0.23
F3H	c51148.graph_c0	96.02	30.11	91.86	45.65
FLS	c34846.graph_c0	132.14	46.77	14.71	296.12
FLS	c15244.graph_c0	0.31	0.36	129.39	104.47
FLS	c39921.graph_c0	2.61	0.94	4.75	3.37
F3'H	c50742.graph_c0	403.64	606.73	54.89	669.55
F3'H	c53714.graph_c0	93.93	1312.82	66.71	27.54
F3'H	c44619.graph_c0	143.04	33.60	92.09	248.54
F3'5'H	c53168.graph_c0	306.56	239.26	41.56	191.94
F3'5'H	c42339.graph_c0	5.06	2.48	2.21	0.38
DFR	c50255.graph_c0	9.03	7.13	340.61	180.64
DFR	c18471.graph_c0	0.03	0.00	6.85	0.78
3GT	c50034.graph_c0	4.13	0.63	67.47	50.36
3GT	c53097.graph_c0	67.93	186.07	56.76	52.79
3GT	c53826.graph_c0	28.88	102.01	173.08	41.52
3GT	c52213.graph_c0	138.37	188.38	107.23	213.52
5GT	c40635.graph_c0	30.19	44.77	10.97	24.56
7GT	c53982.graph_c1	54.24	58.47	77.48	22.84
ANS	c48790.graph_c0	16.64	6.74	854.13	973.91
AOMT	c47583.graph_c0	16.55	0.88	130.32	316.36
ANR	c41299.graph_c0	5.30	0.33	45.66	238.77
PSY	c45574.graph_c0	19.73	124.76	58.31	38.48
PDS	c54027.graph_c0	123.74	381.98	116.08	180.21
Z-ISO	c48628.graph_c0	60.66	55.59	23.60	24.22
ZDS	c51870.graph_c0	128.30	240.23	158.45	116.39

CRTISO	c40622.graph_c0	10.12	24.68	19.87	41.97
CRTISO	c28034.graph_c0	6.07	2.17	4.99	2.06
CRTISO	c53132.graph_c0	11.81	23.31	19.51	17.45
LYCB	c50417.graph_c0	7.65	114.14	58.57	4.21
LYCE	c25556.graph_c0	2.37	1.80	1.63	0.81
CYP97C	c52062.graph_c0	9.14	20.08	13.19	10.23
HYDB	c50756.graph_c0	47.50	254.63	119.95	160.33
ZEP	c36878.graph_c0	12.08	21.12	13.13	3.51
ZEP	c51071.graph_c0	8.14	4.21	8.44	4.06
ZEP	c14141.graph_c0	13.58	3.78	42.86	6.50
ZEP	c49168.graph_c0	42.48	31.58	40.87	13.29
ZEP	c54788.graph_c0	11.05	10.32	15.13	3.78
3GT	c50034.graph_c0	4.125925	0.625916	67.46513	50.3578
3GT	c52483.graph_c0	1.70803	1.08417	2.493024	2.303281
3GT	c39825.graph_c0	4.926925	5.947274	17.68147	4.248357
3GT	c49502.graph_c0	23.27366	6.900665	18.29258	10.96906
3GT	c53097.graph_c0	67.9348	186.0711	56.76495	52.78975
3GT	c31006.graph_c1	0.101864	0.413649	0.836986	0.434904
3GT	c51852.graph_c0	11.016	5.197959	15.02908	27.75712
3GT	c51479.graph_c0	3.565154	0.940088	3.369856	14.37473
3GT	c13285.graph_c0	0	0	2.097314	0.061308
3GT	c17924.graph_c0	0.039159	0.040049	1.552195	0.032272
3GT	c23288.graph_c0	0	1.712085	0.348029	0
3GT	c53826.graph_c0	28.88303	102.0076	173.0828	41.51705
3GT	c52213.graph_c0	138.3747	188.3812	107.2341	213.516

Table S8. Putative unigenes related to transcriptional regulation and transferase activity in *Primula vulgaris*.

**Function**	**Gene**	**Enzyme**	**Unigene number**
Transcriptional regulation	MYB	MYB transcription factor	92
	bHLH	bHLH transcription factor	106
	WD	WD-repeat protein	21
Transferase activity	GST	Glutathione S-transferase	62
	MATE	Multidrug and toxic extrusion transporter	32
	MRP	Multidrug resistance-associated protein	2

Table S9. Three transcription factor families differentially expressed in different-colored petals.

Gene name	Unigene ID	FRKM			
		WL[a]	HL[a]	BS[a]	BO[a]
R2R3-MYB transcription factor	c38443.graph_c0	8.64	43.69	28.46	3.08
	c19192.graph_c0	2.83	0.95	6.57	0.57
	c47013.graph_c0	1.92	7.93	2.83	3.83
	c18654.graph_c0	4.83	1.12	0.87	3.34
	c38938.graph_c0	9.89	10.40	8.62	7.42
	c47450.graph_c0	40.89	34.86	46.32	47.96
	c48241.graph_c0	15.88	10.94	13.46	18.25
	c48106.graph_c0	6.21	34.94	2.79	5.94
	c13821.graph_c0	51.02	56.02	48.22	35.77
	c49814.graph_c0	19.39	18.10	13.66	14.75
	c37362.graph_c0	8.55	8.38	9.23	3.17
	c50767.graph_c0	12.47	30.76	15.19	32.46
	c36030.graph_c0	0.67	1.30	1.58	5.89
	c35729.graph_c0	2.89	3.34	3.78	3.52
	c40864.graph_c0	1.55	16.92	0.37	6.02
	c44135.graph_c0	0.75	0.70	0.94	1.71
	c31460.graph_c0	8.94	6.99	5.32	10.44
	c36093.graph_c0	2.67	0.67	0.75	1.04
	c36034.graph_c0	2.13	5.49	6.19	7.81
	c51154.graph_c0	26.26	25.52	37.88	33.72
	c50771.graph_c0	119.67	120.47	63.99	37.38
	c39429.graph_c0	0.00	0.62	0.00	0.77
	c53328.graph_c2	11.68	13.92	14.34	17.60
	c45347.graph_c1	13.57	11.70	0.80	15.12
	c47527.graph_c0	12.77	12.24	13.05	10.42
	c49783.graph_c0	33.31	47.85	45.31	32.36
	c38596.graph_c0	7.12	14.18	11.93	1.22
	c36140.graph_c0	3.48	1.11	1.71	2.04
	c39130.graph_c0	10.13	16.01	11.09	11.37
	c48646.graph_c0	149.88	71.82	60.90	110.42
	c41292.graph_c0	18.49	8.96	7.25	10.53
	c28376.graph_c0	11.83	20.79	16.88	18.66
	c47997.graph_c0	5.05	7.25	7.04	4.09
	c52398.graph_c0	2.00	3.88	1.73	3.57
	c52151.graph_c0	17.83	23.17	20.56	10.30
bHLH transcription factor	c32022.graph_c0	1.86	1.48	2.27	1.45
	c19199.graph_c0	0.00	7.43	9.48	0.02
	c45036.graph_c0	11.33	9.41	12.66	8.69
	c37160.graph_c0	1.08	1.09	2.14	1.21
	c13543.graph_c0	0.10	0.09	1.51	5.59
	c43431.graph_c0	10.85	10.50	7.60	9.06

	c46449.graph_c0	3.09	11.92	2.44	8.04
	c42896.graph_c0	6.66	6.21	9.23	3.44
	c44399.graph_c0	12.59	13.64	18.06	15.51
	c54293.graph_c0	5.91	6.29	5.38	4.56
	c51897.graph_c0	8.66	7.59	8.22	8.10
	c45582.graph_c0	1.13	1.42	1.03	0.51
	c41723.graph_c0	12.36	15.78	8.16	9.23
	c51041.graph_c0	1.76	0.54	0.45	0.49
	c52581.graph_c0	6.69	8.77	8.64	5.23
	c34305.graph_c0	1.55	0.57	1.48	1.09
	c51557.graph_c0	7.02	4.93	3.26	3.82
	c55405.graph_c0	6.85	11.87	6.55	8.10
	c50758.graph_c0	1.17	8.28	1.18	1.69
	c49536.graph_c0	1.59	11.22	2.39	2.21
	c50788.graph_c0	15.40	17.19	15.68	14.10
	c53803.graph_c2	26.19	9.63	18.28	27.98
	c18795.graph_c0	5.71	11.14	4.83	8.16
	c45751.graph_c0	1.08	0.39	1.33	3.79
	c52273.graph_c0	5.39	32.32	13.76	10.73
	c42402.graph_c0	6.53	0.07	0.47	3.12
	c52537.graph_c0	19.50	35.19	24.05	28.74
	c52918.graph_c0	143.68	95.11	100.04	139.67
	c36176.graph_c0	1.63	2.99	8.30	0.37
	c18047.graph_c0	0.00	0.02	0.52	1.03
	c44173.graph_c0	2.37	0.26	1.28	2.93
	c50718.graph_c0	9.17	19.65	8.34	16.87
	c52261.graph_c0	15.09	17.48	5.30	5.62
	c54481.graph_c0	3.24	4.83	1.92	4.25
	c53831.graph_c0	14.70	12.36	16.40	17.50
	c43227.graph_c0	7.20	6.05	8.98	8.79
	c42257.graph_c0	2.74	3.80	3.81	2.80
WD40- protein	c35345.graph_c0	13.13	15.97	32.73	10.27
	c41101.graph_c0	5.63	7.07	8.06	7.00
	c49478.graph_c0	10.42	8.66	7.43	6.95
	c54879.graph_c1	20.24	35.09	19.93	16.00
	c43450.graph_c0	16.63	20.69	15.01	19.43

Table S10. List of primers used for qRT-PCR analysis.

Gene	Unigene ID	Forward primer(5'-3')	Reverse primer (5'-3')
GFP	GFP	CAAACCAAAGAATGGCATCA	AAAGGGCAGATTGTGTGGAC
PAL-1	c46806.graph_c0	GAGGCGTTTAAGTTAGCGGG	AGCCGATAAAACCTCCGACA
PAL-2	c48032.graph_c0	CGACACAAAGACTGCCAACA	AAAACCCAGTCGCCAATTCC
CHS-1	*c*34457.graph_c0	TGGTTACTGTCGAGGAAGTCC	ACATGCGCTTGAATTTCTCCT
CHS-2	c14043.graph_c1	TCGATCAGAGCACGTATCCA	CTAGCGTCAAATGAAGGCCC
CHS-3	c36929.graph_c0	ACAATGATGGTGCACGCTTT	AAAATCATCCCCTCACCGGT
CHS-4	c43007.graph_c0	GAGGCAAAGCTTGGGTTGAA	TCTACAGTCAAACCCGGTCC
CHS-5	c14043.graph_c0	GCCGAAAACAATAAAGGCGC	GTCTGGGCCGCAGATACTAT
CHS-6	c57559.graph_c0	CAACGGAAAGGACCAAGGTG	GGGGAAAGTAGGCTGGGATT
CHS-7	c53024.graph_c0	TGTCAAACGCGTCATGTTGT	GCTTCTCGACGTCAGGGATA
CHS-8	c41877.graph_c0	ACCTGATGATACGAGGGCTG	CTGATTCTGGGACGAGGGTT
CHS-9	c46201.graph_c0	AACTCACTAAGCTGCTCGGT	ACGGAAGGTGACAGCAGTTA
CHI-1	c27920.graph_c0	GAACATGTCTTCCCAGCGAC	TCGGTCAATTCCTCCTTGCT
CHI-2	c40107.graph_c0	GTTTCTGCGAGTTGTGGTGA	GGTGGAGGTAGCAGGGAAAT
FLS -1	c34846.graph_c0	AAATCGGGTGAAGGAGGGAG	CAACCTTCCTGCCACTTTCC
FLS-2	c15244.graph_c0	TCATGAACGTCCCGAGAACA	CGATCGATCAACTCTTGCGG
FLS-3	c39921.graph_c0	AAGGAGATCGGTGGGCTTAC	CTTTGATCACGTGAAGGCCA
F3H-1	c82473.graph_c0	CCAGCCACTTACAGGGAGAA	TGCTTCCTTCTCCAATCCCA
F3H-2	c48030.graph_c0	CTCCTGAGGGTTATGGCCAA	CAGACCCTGCACTTCATTGG
F3H-3	c43100.graph_c0	GACCGTCCAAACAGCAATGT	TTTAGACGCTCGCTCTCAGG
F3H-4	c51148.graph_c0	GCCTTGACAGTTCTTGCACA	GCTGGGTTGAAGAAGAACGG
F3‘H-1	c53714.graph_c0	TGGTTTTCTGTCAATGGCGT	GGCACAAAATTTACCCCAGC
F3’H-2	c50742.graph_c0	AGAGGCTCAACGACACTGAA	AAGTCGATTTGGGCCCACTA
F3'H-3	c44619.graph_c0	GGGTTCTTGGACTTGCAAGG	TTGACACCGTGCCTTTCAAG

DFR-1	c50255.graph_c0	TGCTCATCACACGAAGCAAC	AGAGGAGAAGGGGAGCAAAC
DFR-2	c18471.graph_c0	TGAGAGCGACTGGTGTGATT	TGGCCCTCGTTTCCTGTAAT
F3'5'H-1	c53168.graph_c0	CCCAACAACGCCCTTAAGTC	TCTTTTCCCACCGCCAAATG
F3'5'H-2	c42339.graph_c0	GCCGAATCTCCCATACCTCA	ATGTTGGCTGTCTCTCCTCC
ANS-1	c48790.graph_c0	CTAGGCCCACAACTCCCTAC	CCCAGAAGCTTGATCGTTCG
ANR-1	c41299.graph_c0	CGAGTTTGGGCACGACATAG	GGGTGGGTATTGTCGTGGTA
AOMT-1	c47583.graph_c0	TGGCATGTCTAGCTCTCCTG	AGCCTCACTTGCAATGAAGC
3GT-1	c50034.graph_c0	TTCCTTTCGGTACCCATGCT	ACCGCCTCTCTAAACCCTTC
3GT-2	c53097.graph_c0	ACCAGCCTCCTTCTTCTGTC	CGTAGTCAGTAGTGGGTCCC
3GT-3	c53826.graph_c0	ACGTGGCCCTTATATGCTGA	TCCCTCTAATGCCCGAACAA
3GT-4	c52213.graph_c0	CCCCTACACCGAGACTCTTC	CACTCCCAACTCACTAGCCA
5GT-1	c40635.graph_c0	AGATGTTTGGGAGATGGGTGT	AATGACGACCCACCTTCCTT
7GT-1	c53982.graph_c1	ACCGGTAGTGTCGTTTTCCA	ACGAGGCCTCCGATTAATGT
THC2'GT-1	c53097.graph_c0	ACCAGCCTCCTTCTTCTGTC	CGTAGTCAGTAGTGGGTCCC
THC2'GT-2	c53826.graph_c0	ACGTGGCCCTTATATGCTGA	GCATTGTAAGAGGATCCGCC
THC2'GT-3	c52213.graph_c0	ACGTATGCATTTCGTCAGCC	CACTCCCAACTCACTAGCCA
4CL-1	c54775.graph_c1	ATTCCTTTGCCCAAACCCAC	AGAGGTCCGAAACTGCTTGA
LYCB-1	c50417.graph_c0	GCTAGGTTGAGGCATTTGGG	GCATTGGCCAGAATAGGAGC
HYDB-1	c50756.graph_c0	CGACTCATTACCTCCGGCTA	GCTACTTTCTCTGCCACGTG
PSY-1	c45574.graph_c0	TATTACGTGGCTGGAACCGT	ACACTCTTCCCCTTCTTGCA
ZDS-1	c51870.graph_c0	TGTACGTTGCAGCTTGTGAC	TAGTTGCCTTGCCCGTTCTA
CRTISO	c53132.graph_c0	CAAGGGGCGCCATATTCTTC	CAGAAGACTTGAGCCCTGGA
CYP97C-1	c52062.graph_c0	TTGGGTCTGGGTTCGCTATT	TAGGCCTATCACATCGAGCG
PDS-1	c54027.graph_c0	TGTGTTTGCAACTCCAGTCG	CAAGCTACGGACATGTCAGC
ZEP-1	c54788.graph_c0	ACCCCAGACGACACAATCAT	AACTCGCCATTCTTAACCGC
GST-1	c41154.graph_c0	TTTTGCCAACCGGATCCAAG	GGGAAGGATAGAAGGACCGG

GST-2	c41828.graph_c0	TGAACAGAGCCCAAATTGCC	AGAATAGTAGGACCCGCTGC
GST-3	c41235.graph_c0	CATCTGGCAAGCTCCTTTGG	TACGCTAGATTGGGGAGGTG
MATE-1	c38972.graph_c0	CAAGCTCACTGTTGCCTCTG	TGCCCCTAGTTCATTGCTCA
MATE-2	c43565.graph_c0	GTAACACTGTCAATGCCCCA	TCACAAATGACCACGGCAAT
MATE-3	c50798. graph_c0	CCAAACTCGCAGCTCTGTAC	CGGCCCCTTAAAACCAAGAG
MYB-1	c44135. graph_c0	GCCATGGAGTGCTGATGAAG	GACCACGCTTAATATCCGGC
MYB-2	c40864. graph_c0	TCGGTGCATGGATTCAGAGG	TCGTCTTCATCCATAGCCCA
MYB-3	c36140.graph_c0	TTGGGACTATGCCACCACTT	GTGGCCAATCTAAGCTGCAA
MYB-4	c19192.graph_c0	TCGTGTCTACCCGGAAGAAC	GGTTTCTAGCTGGGGAGAGG
MYB-5	c47013.graph_c0	TAGATGTGACGTCGGGGATG	ACCCTTGTAACCCCGATCTG
MYB-6	c18654.graph_c0	TGACGCCAGATACACCAACT	TCCAAATCCCAGTCCCAGTC
MYB-7	c13821.graph_c0	GCACTTTTGGCTCGAAGACA	AGAGTGGTTGTAGTGAGGGC
MYB-8	c49814.graph_c0	TGAACATGAGCCCTACTGCA	GCGACAAAGGCGAAAGATCT
MYB-9	c49783.graph_c0	TGGAACCCTCTGCAGATGTT	CTTTCACTGCCACCAAGCTT
MYB-10	c39130.graph_c0	TCGGGCCATGACCAAGTATT	CCCAGATGATCTTGCAACCG
MYB-11	c45347.graph_c1	CGCGGATGAGATCAAAACGA	CCCAAGCTGCAAGAGAAACA
bHLH-1	c52273.graph_c0	GGACGAGGAAAATGACGACG	TGACGTTGGTGTAGACTCCC
bHLH-2	c50718.graph_c0	AAGATTACGGGGAAGGCTGT	ATCGTCACATTGCCACTTCG
bHLH-3	c52261.graph_c0	CCGGGTTGTAACAAGGTGAC	GATGAATCGGCTCGGTATGC
WDR	c43450.graph_c0	AGTTCAGCCTGCAGTGGTAT	TGGGCCATGGTAATAGTGCA
actin	c55882.graph_c0	GGCAGTGTTCCCTTCCATTG	TCCATATCGTCCCAGTTGCT

Table S11. List of primers used for gene cloning.

Gene	Unigene ID	Forward primer(5'-3')	Reverse primer (5'-3')
MYB-1	c44135. graph_c0	GGGTACCATGGGGAGGACACCATGT	CCCCGGGATAATCATATACACGCT
MYB-2	c40864. graph_c0	GGGTACCATGAGAAAACCATGTTGT	CCCCGGGACTGAACAGAAAAAGTG

Renfernece:

1. Harborne, J.B. Comparative Biochemistry of Flavonoids-VII Correlations between Flavonoid Pigmentation and Systematics in Family Primulaceae. *Phytochemistry* **1968**, *7(8)*, 1215-1230.

2. Venter, A.; Joubert, E.; de Beer, D. Characterisation of phenolic compounds in South African plum fruits (*Prunus salicina* Lindl.) using HPLC coupled with diode-array, fluorescence, mass spectrometry and on-line antioxidant detection. *Molecules*, **2013**, *18(5)*, 5072-5090.

3. Harborne, J.B. Plant Polyphenols-XV Flavonols as Yellow Flower Pigments. *Phytochemistry* **1965**, *4*, 647-657.
